# Theoretical demonstration of a capacitive rotor for generation of alternating current from mechanical motion

**DOI:** 10.1038/s41467-021-23891-6

**Published:** 2021-06-16

**Authors:** Ehud Haimov, Aidan Chapman, Fernando Bresme, Andrew S. Holmes, Tom Reddyhoff, Michael Urbakh, Alexei A. Kornyshev

**Affiliations:** 1 School of Physics and Astronomy, Raymond and Beverly Sackler Faculty of Exact Sciences, Tel-Aviv University, Tel-Aviv, Israel; 2 Department of Chemistry, Faculty of Natural Sciences, Imperial College London, Molecular Sciences Research Hub, White City Campus, London, UK; 3 Thomas Young Centre for Theory and Simulation of Materials, Imperial College London, South Kensington Campus, London, UK; 4 Department of Electrical and Electronic Engineering, Faculty of Engineering, Imperial College London, South Kensington Campus, London, UK; 5 Department of Mechanical Engineering, Faculty of Engineering, Imperial College London, South Kensington Campus, London, UK; 6 School of Chemistry, Raymond and Beverly Sackler Faculty of Exact Sciences, Tel-Aviv University, Tel-Aviv, Israel

**Keywords:** Devices for energy harvesting, Electrical and electronic engineering, Mechanical engineering, Electrochemistry, Electronics, photonics and device physics

## Abstract

Innovative concepts and materials are enabling energy harvesters for slower motion, particularly for personal wearables or portable small-scale applications, hence contributing to a future sustainable economy. Here we propose a principle for a *capacitive rotor device* and analyze its operation. This device is based on a rotor containing many capacitors in parallel. The rotation of the rotor causes periodic capacitance changes and, when connected to a reservoir-of-charge capacitor, induces alternating current. The properties of this device depend on the lubricating liquid situated between the capacitor’s electrodes, be it a highly polar liquid, organic electrolyte, or ionic liquid – we consider all these scenarios. An advantage of the capacitive rotor is its scalability. Such a lightweight device, weighing tens of grams, can be implemented in a shoe sole, generating a significant power output of the order of Watts. Scaled up, such systems can be used in portable wind or water turbines.

## Abstract (web summary)

Energy harvesting devices based on micro-electromechanisms (MEMS) is attractive for sustainable energy applications. Here, the authors report the theoretical working principle of a lightweight, low-voltage AC-current generating MEMS-based capacitive rotor device for Watts-level power generation from everyday walking.

## Introduction

In today’s world, people are increasingly finding themselves carrying several battery-powered portable devices such as mobile phones, laptops, and smartwatches. Typical usage of these devices involves the batteries being fully depleted and then recharged with mains electricity or portable power kits. This electricity is still usually sourced from non-renewable resources, such as fossil fuels, despite suggestions that 100% renewable energy systems are both technologically and economically feasible^1^. This issue is exacerbated by the number of such battery-powered devices in operation. In an ideal world these portable devices would have their own internal green power source without the need of external recharging.

Current research into the field of energy harvesting is focused on harnessing the low-frequency mechanical energy that is ordinarily dissipated as heat and converting it to useful electrical energy, in small (cm- or mm-scale) devices^2–6^. Potential sources of this mechanical energy include human body motion, machine motion/vibration, and fluid flow. The same principle is used by conventional renewable energy sources that convert solar, wind, or thermal energy. Energy harvesting by electromagnetic (EM) induction from vehicular motion and the mechanical vibrations of heavy machinery are both highly developed, the former being the basis of regenerative breaking. There are, however, only a limited number of systems to date that take advantage of the power we generate whilst walking or performing any other slow motion. This new generation of devices can be implemented in wearable systems to offer the promise of autonomous generation of energy for personal use. Indeed, some systems have already seen implementation into the consumer market, for example in kinetic watches.

EM dynamos that use low-frequency motion to generate current are ordinarily far too bulky to be used for personal wearables. Millimeter-scale EM-based energy harvesters have been demonstrated, but these are typically narrow-band devices with center frequencies significantly higher than the typical frequencies associated with human movement. For example, the device reported in^7^ had a peak output power of 46 μW when excited at 52 Hz and a bandwidth of ~2 Hz. The power generated by a walker with such device would be at the sub-μW level. Recent higher power devices of this class include one based on a cycloid shaped tube containing a magnetic ball wrapped in wire coils, that can be worn around the arm or leg of a runner^8^. The motion of the ball through the tube induces a current in the wires. The device has a diameter large enough to fit around a wrist and can generate ~9 mW when excited at 5 Hz^8^. This level of power is potentially useful for supporting a limited range of biometric sensors, but not for recharging a mobile phone.

EM transduction does not scale particularly well to small dimensions, and consequently much of the research to date in mechanical energy harvesting has been focused on alternative energy conversion mechanisms, particularly those that can be implemented using micro-electromechanical systems (MEMS) and nano-electromechanical systems technologies^7^. These technologies allow the realization of miniaturized energy harvesters based on a range of physical effects including piezoelectricity^9^, triboelectricity^10^, and electrostatics via capacitive charging^11–13^. Devices based on these effects can potentially harness the electrical power from slow but large amplitude motions related to human walking.

A key challenge when harvesting power from low-frequency motion with a miniature energy harvester is that of achieving sufficiently strong electrical damping in the electromechanical transducer. A common approach is to employ frequency up-conversion whereby the slow-moving proof mass excites a higher-frequency vibration in the transducer. In piezoelectric devices this is typically done by plucking a piezoelectric beam once (or several times) per cycle of the proof mass motion and allowing it to ring down at its natural frequency^2,3^. In this way the elastic energy stored in the beam each cycle is converted to output power (rather than moving back and forth between the beam and the proof mass). The same frequency up-conversion principle has been used to enhance the power generation capability of EM-based energy harvesters^5^.

Piezoelectric devices are perhaps the oldest and most straightforward class of MEMS energy harvesters. In a piezoelectric material, stress causes distortions in its structure, polarizing it spontaneously, which results in a transient electric current. A simple piezoelectric energy harvester consists of a piezoelectric material sandwiched between two metal electrodes. By applying a mechanical strain, a piezoelectric polarization occurs. Releasing the strain decreases the polarization and consequently current must flow in the opposite direction. This principle was used in piezoelectric shoe soles as early as 2001^14^, but with the particular design investigated the power generated from walking frequencies was limited to around 1 mW.

The triboelectric effect^15,16^ is a well-known physical phenomenon in which static charges of opposite sign and equal magnitude are formed at the interface between two dissimilar insulating materials moving in close contact. Rubbing the materials together results in an enhanced effect as they come into contact multiple times. The essence of this effect is the static electricity that we encounter in day-to-day life, but in the corresponding MEMS devices it is utilized in generation of useful alternating currents. The resulting formation of a dipole across the interface produces an interfacial electric field and a voltage drop between the interfaces. Exploitation of triboelectrically generated voltage in energy harvesting devices was pioneered and systematically studied by Wang et al.^17–19^. In these devices, two dissimilar polymer films backed with gold electrodes, in electrical contact, create an interfacial potential difference, which induces current flow between the two electrodes. All triboelectric generators based on the contact of solid surfaces are prone to gradual degradation and failure after a prolonged use. The scalability of these generators is limited by the surface area of the contact, the number of paired contacts that can be accommodated, and the maximum surface charge density that can be generated between the materials in contact.

Reverse electrowetting devices (REDs) overcome many of these limitations. Electrowetting is a process by which a droplet of liquid containing an electrolyte may be spread on a charged surface, with the extent of wetting being controlled by the applied voltage. The reverse electrowetting, conversely, involves mechanically changing the extent of wetting which changes the electrical capacitance of the system. This can be achieved by placing a non-wetting or partially wetting liquid droplet between two electrodes kept at a constant voltage and then forcing the droplet to spread beyond its natural contact angle by compressing it between the electrodes. This will trigger a flow of charge between the electrodes, in order for each of them to attain the charge that satisfies the new equilibrium value of capacitance. Using electrolytic solutions utilizes the capacitance of electrical double layers at electrodes, making the capacitance much higher than obtained with pure dielectric liquids, and determining the change of capacitance to essentially the change of the contact area of the droplets with the electrodes.

Krupenkin et al.^11,12^ first introduced a RED energy harvester in 2011, with both experimental results and a theoretical analysis of the proposed system. Krupenkin and Taylor^12^ considered electrolytes sitting on top of a flat electrode and one of a variety of proposed mechanisms by which a periodic pressure is applied to the droplet array. This periodic pressure can be achieved by placing the droplet array between oscillating or shearing plates. Krupenkin and Taylor reported high power densities of up to 1 kW m^−2^. In an alternative device structure, they placed the droplets in channels with walls containing patched regions of dielectric and metallic materials. By applying a periodic pressure at the end of a tube, the droplets move between the regions of dielectric and metal and thus the contact area with the metallic electrode materials periodically changes, resulting in variation of capacitance and thus in generation of electrical current.

Later theoretical works by Kolomeisky and Kornyshev^20^ and the follow up by Kornyshev^21^ et al. considered the utility of reverse electrowetting harvesters with porous electrodes. The system proposed there consists of an electrode with many cylindrical pores of micron or submicron radius, with the pores much longer than they are wide. The electrode is coated with or made out of a material that is solvophobic to the ionically conducting liquid bulk which sits on top of the electrode^21^. In this way the capillary pressure prevents the conductive liquid from entering the pores without the application of an external pressure. The advantage of this system is the availability of micro to nanoporous carbon materials with volume filling pore space. Such porous materials allow for a many orders-of-magnitude increased accessible surface area for the electrolyte under applied pressure, thus allowing for more energy to be transferred per cycle. But this system has not been yet experimentally realized.

Both the Krupenkin^11,12^ and Kolomeisky and Kornyshev^20^ devices can potentially suffer from hysteresis of the spreading of the droplets. Once the mechanical strain is released, will the droplets fully return to their original shape? If the answer to this question is no, then the change in capacitance over a cycle becomes lower, weakening power output. This problem is exacerbated in the porous system, as it becomes harder for the liquid to escape out of the pores in time with the pressure release. This difficulty can, in principle be mitigated by engineering, through generation of the gas pressure from the other side of the porous electrode, in order to release the liquid from the pores back to the solution as needed.

These RED devices belong to a more general category of capacitive energy harvesters, in which applied mechanical work changes the capacitance of the device, in turn inducing flow of the charge, i.e., generating transient electric currents to re-balance the charge on the electrodes subject to the changed capacitance. Alternating the capacitance periodically results in AC currents.

In the above examples, capacitance was varied by squeezing liquid droplets to change their contact area with electrodes. In more recent examples, capacitance was varied instead by changing the area of submersion of the electrodes in an electrolyte^22^ or sliding electrodes relative to each other^23^.

In this paper we propose a different principle for a current generating MEMS device. One of its main features is that it does not involve the change of the liquid contact area, as the electrodes remain permanently immersed in the liquids. It is thus based on continuous, hysteresis-free cyclical alteration of the electrode configuration through rotation of the electrode. Other attractive features of this setup are that it (1) is expected to minimize hydrodynamic issues (such as turbulence), (2) results in low friction and, unless deformed, no surface wear, (3) and when carefully designed might not need to address specific heat management. At the same time, it delivers, as we will show, significant power and can be easily scaled up. Energy harvesters based on this principle may be used not only within “electrical shoes,” but also, in portable, alternating current generating mini-turbines driven by wind or water streams. Our proposed device has advantages over triboelectric and piezoelectric devices in terms of operational lifetime. Triboelectric generators based on the contact of solid surfaces are prone to degradation and failure after prolonged use as a result of wear^24^. Piezoelectric energy harvesters are also prone to degradation over time because cycling the piezoelectric element to the required high strain levels could lead to the development of micro-cracks^25^. The power density of our proposed device will be higher than that of other capacitive energy harvesters because we have a dielectric in the variable capacitor rather than an air gap. The higher permittivity of the dielectric will increase the energy converted per cycle, all other things being equal. The current generating systems proposed here has yet to be built and tested. However, we see no fundamental problems with their realization, and present below a detailed theoretical analysis of its properties and functional characteristics, which will assist in the design of this new type of MEMS-based converters.

## Results

### Working principle

**Fig. 1:**
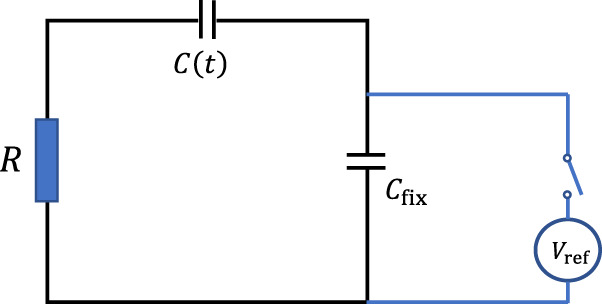
A schematic for the electric circuit of the device. A capacitor of fixed capacitance ${C}_{{\rm{fix}}}$ is connected in series to a time-varying capacitor, $C\left(t\right)$, and an electric load, $R$. The capacitor ${C}_{{\rm{fix}}}$ is initially connected to a priming voltage source ${V}_{{\rm{ref}}}$ for momentary charging and is then disconnected.

Consider the electric circuit shown in Fig. 1. It contains two capacitors, one of which is pre-charged and has a fixed capacitance ${C}_{{\rm{fix}}}$ while the other possesses a time-modular capacitance $C\left(t\right)$. The capacitors are connected in series together with an electric load of resistance $R$. If the system is left by itself, then eventually, after enough time, the electric charge will redistribute between the two capacitors, so that the difference in the voltage between them becomes zero. A change in the voltage difference between the two capacitors can then be achieved by investing mechanical energy to change the capacitance of the time-varying capacitor. Subsequently, an electric current is generated, flowing through the resistor, to level the voltage difference between the two capacitors. Repeating this process periodically would generate an alternating current through the circuit. To enhance this effect, we consider the fixed capacitance ${C}_{{\rm{fix}}}$ to be much larger than the time-varying capacitor $C\left(t\right)$. Thereby, the fixed capacitor ${C}_{{\rm{fix}}}$ serves as a reservoir of charge.

The principle of operation of such device is well illustrated by the following mechanical analogy. A water reservoir and a modularly water vessel are connected by a pipe. Inside the pipe there is a turbine that can freely spin in any direction subject to the direction of water flow. According to Pascal law, the heights of the two communicating vessels will level after some time. Squeezing or expanding the modular vessel will initially result, respectively, in the increase or decrease of water level inside it. Then, due to the height-level difference, a current of water will flow, respectively, from the modular vessel to the reservoir or back, spinning the turbine in the process, each time in opposite direction.

As will be shown in the “Theory” section, the power on the resistor will practically be independent of the value of reservoir capacitance, ${C}_{{\rm{fix}}}$, but the power will increase with the average magnitude of $C\left(t\right)$. In order to increase the total time-varying capacitance, we consider a device containing $N$, synchronized time-varying capacitors, ${C}_{1}\left(t\right)$, all connected in parallel to give a total time-varying capacitance of:

1 $$C\left(t\right)=N{C}_{1}\left(t\right).$$

**Fig. 2:**
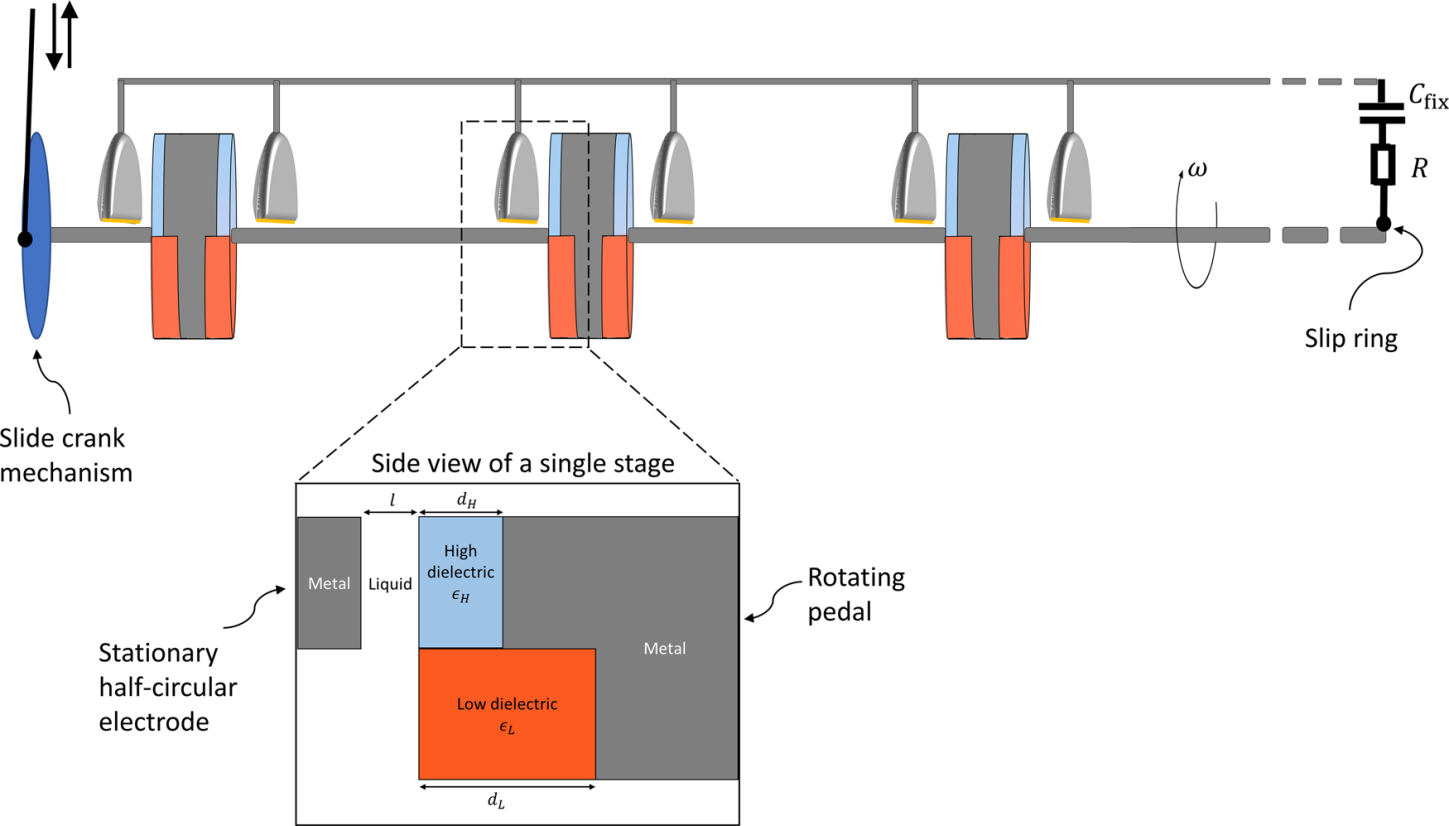
Illustration of the energy harvesting device. The device comprises of a multi-stage rotor structure, connected to a reservoir capacitor ${C}_{{\rm{fix}}}$ and to an electric load $R$. The design of the rotor structure is presented (not to scale) with an additional side view of a single stage which consists of a rotating disc and a stationary half-circular metal electrode; in the gap between the two is a lubricating liquid. Each rotating disc is shared by two adjacent stages, connected in a back-to-back fashion, in order to save space, reduce the overall mass and prevent electric shortcuts. All the stages are mounted on a common metal axle, thereby effectively connecting all stages in parallel. To ensure the prevention of shortcuts between the metal axle and the bottom of the half-circular electrode, the bottom should be insulated, as depicted by a thin yellow line. The rotor can be spun by different means, in the figure we show one option of a slide-crank mechanism, however it can equally be rotated by a wind- or water-turbine. Mechanical components to optimize the shaft rotational velocity (gearset, freewheel, and flywheel) have been omitted from the illustration for clarity.

The scheme for a single time-changing capacitor ${C}_{1}\left(t\right)$ is illustrated in Fig. 2. The capacitor is composed of a stationary semi-circular metal electrode and a disc which is part dielectric and part metal. The gap between the disc and the semi-circular electrode is filled with a lubricant. Two different dielectric materials, with different thicknesses, are situated at each half of the disc. Thereby, rotation of the disc will generate a change in capacitance (see “Theory” section). This design protects the system from electrical short-circuits that would appear by physical contact between the disc and the semi-circular electrode. The dielectric material, which faces the semi-circular electrode, is an electric insulator, and therefore prevents the short-circuit.

To synchronize the operation of $N$ such capacitors, connected in parallel, we devised a scheme where all discs rotate together on a single metal axle. The metal part of each disc is then kept at the same electric potential, and the semi-circular electrodes are all connected, thereby putting them at a different joint electric potential (see Fig. 2). In this paper we consider up to ${10}^{3}$ stages on a single axle. However, an even larger number of repeating units may also be distributed over a few separate axles, synchronized together by connecting the different slide-crank wheels.

The axle can be rotated by various means, such as by a wind- or water-turbine or by a periodic press on a slide-crank mechanism. Here we will mainly consider a slide-crank mechanism (see Fig. 2), operated by a periodic press, such as the pressing of foot in the process of walking.

### Theory

A single capacitor is composed of two metal electrodes, one which is part of the rotating disc, and the other which is immobile. Between the two electrodes are dielectric materials, coated onto the disc’s metal electrode and a lubricating liquid in the gap between the disc and the immobile half-circular electrode.

Each half of the disc has a different dielectric material, each with its own thickness. When the disc rotates, the dielectric structure between the electrodes changes, thereby causing a continuous and periodic change in capacitance between a minimal value ${C}_{{\rm{min }}}$ and a maximum value ${C}_{{\rm{max }}}.$

### Changing capacitance—estimates within a simplified model

To enhance the effect, we need to maximize the change in capacitance during the operation cycle. To that end, we designate ${\epsilon }_{H}$ and ${\epsilon }_{L}$, the high and low dielectric constants of the different dielectric materials, to have as high as possible and as low as possible values correspondingly. For the current setup we considered titanium dioxide and Teflon as the high and low dielectrics having dielectric constants of ${\epsilon }_{{\rm{H}}}\approx 128$ (the dielectric response of TiO_2_ is anisotropic, and for simplicity we take this average value for the numerical analysis) and ${\epsilon }_{{\rm{L}}}\approx 2$, respectively^26^. To further enhance the change in capacitance, we considered different thicknesses for each of the dielectric materials. The difference in capacitance is greatest when the thickness of the high-dielectric material, ${d}_{{\rm{H}}},$ is as small as possible, ideally just a few nanometers, generating the greatest maximal capacitance ${C}_{{\rm{max }}},$ while the low dielectric material thickness, ${d}_{{\rm{L}}}$, is as high as needed to achieve minimal capacitance ${C}_{{\rm{min }}}\ll {C}_{{\rm{max }}}$. The values for ${C}_{{\rm{min }}}$ and ${C}_{{\rm{max }}}$ correspond to the configurations in which the low and high-dielectric elements are in front of the immobile half-circular metal electrode. Hereafter, we considered discs of 1 cm radius coated with dielectric films of thicknesses *d*_L_ = 200 nm and *d*_H_ = 5 nm.

The properties of the lubricating fluid in the gap between the disc and the immobile electrode have a high impact on the efficiency of the device. The fluid’s electrical properties, i.e., its screening length and dielectric constant greatly affect the values of ${C}_{{\rm{min }}}$ and ${C}_{{\rm{max }}}$. The lubricity of the fluid affects how fast the disc can spin, thereby setting the rate of capacitance variation. The lubricant must be nonvolatile (i.e., have a low vapor pressure at the working temperature) to keep the integrity of the device. The lubricant can either be an ionic liquid (IL) or a low volatility molecular liquid. Lubricants such as perfluoropolyether, which are used in extreme lubrication applications, have a too low dielectric constant, and therefore cannot be readily employed in the device presented in this work. Instead, ILs feature large capacitance even when the gap distance, $l$, between electrode and disc is large on the scale of the screening length, since the potential drops rapidly within a very short average screening distance of about 1–5 nm in the vicinity of each electrode. On the other hand, a molecular liquid lubricant may also provide high capacitance, but only when the gap size, $l$, between the electrode and disc, is extremely small, on the scale of a few nanometers. In both cases, the capacitance increases with the dielectric constant of the fluid, $\epsilon$. The radius of the half-circular electrode and disc, of the order of centimeters, is much larger than the thicknesses of the dielectric layer and the thickness of liquid gap considered here (up to 10 μm), and therefore edge effects can be neglected.

To derive an analytical formula for the capacitance of a single capacitor, we considered the case of an electrolyte liquid as a lubricant. On the one hand, an electrolyte solution provides an exponential decay in the electric potential and electric field, which roughly mimics the screening effect of ILs (neglecting in a simple estimate layering and over-screening^27^), and on the other hand it gives an analytical result that includes the case of molecular liquids as the limiting solution for large screening lengths (larger than the gap between the rotating and immobile electrodes). In the case of electrolyte solution with Debye length $\lambda \equiv {\kappa }^{-1}$ and dielectric constant $\epsilon$, the minimum and maximum capacitance values of a single capacitor are derived in Supplementary Note 1 and given by:

2A $${C}_{{\rm{min }}}=A{\left({\widetilde{C}}_{{\rm{liquid}}}^{-1}+{\left(\frac{{\epsilon }_{0}{\epsilon }_{{\rm{L}}}}{{d}_{{\rm{L}}}}\right)}^{-1}\right)}^{-1}$$

2B $${C}_{{\rm{max }}}=A{\left({\widetilde{C}}_{{\rm{liquid}}}^{-1}+{\left(\frac{{\epsilon }_{0}{\epsilon }_{{\rm{H}}}}{{d}_{{\rm{H}}}}\right)}^{-1}\right)}^{-1}$$

where ${\epsilon }_{0}$ is the permittivity of vacuum, $A$ is the surface area of half-circular electrode and ${\widetilde{C}}_{{\rm{liquid}}}$ is the liquid’s surface capacitance density. Within the simplified model of electrolyte solution, the liquid’s capacitance surface density is given by:

3 $${\widetilde{C}}_{{\rm{liquid}}}=\frac{{\epsilon }_{0}\epsilon \kappa }{2}{{\coth }}\left(\frac{\kappa l}{2}\right)$$

Two distinct designs for the rotor stem from two limiting cases of Eq. (3). The first corresponds to molecular liquids in narrow gaps, for which $\kappa l\ll 1,$ and the second case corresponds to a lubricating liquid with a short screening length in wide gaps, for which $\kappa l\gg 1$.

It should be noted that while the maximum capacitance ${C}_{{\rm{max }}}$ is bounded from above in a nontrivial way, the minimum capacitance, ${C}_{{\rm{min }}}$, in both setups, can effectively be made as small as we like by increasing the thickness of the low dielectric coating ${d}_{{\rm{L}}}$.

To increase the maximum capacitance for a molecular liquid, it is imperative to use a liquid with a high-dielectric constant along with a gap that is as narrow as possible, i.e., in the nm range. We consider here two different high-dielectric and nonvolatile molecular liquids: formamide, of bulk dielectric constant^26^ $\epsilon =111$, and propylene carbonate (PC), with bulk dielectric constant of^26^ $\epsilon =66$.

### Molecular dynamics simulations

The analytical expression for the liquid’s surface density capacitance, ${\widetilde{C}}_{{\rm{liquid}}}$, as given by Eq. (3), is useful for elucidating the key parameters that govern the capacitance value of a single stage capacitor and for obtaining rough numerical estimates for this capacitance. It is, however, not accurate for the analysis of generator performance. The electrical properties of a molecular liquid under confinement conditions might differ significantly from those of the bulk liquid^28^. Also, the screening of ILs is, in reality, much more complicated than that described using the simple linearized Debye screening approximation, both for electrolyte solutions or ILs in the nanogaps. We therefore performed molecular dynamics (MD) simulations to calculate the capacitance and dielectric properties of the two molecular liquids, PC and formamide, confined in a nanogap of width 5 nm in a nanoconfinement space and the capacitance of an IL in a gap wider than its screening length. Eventually, the overall capacitance of a single stage will be calculated by connecting in series the lubricated layer capacitance density ${\widetilde{C}}_{{\rm{liquid}}}$, as acquired from molecular simulations, together with the capacitance of the dielectric layer, as suggested by Eqs. (2A, B).

### Simulation of a nanogap capacitor filled with propylene carbonate and formamide

We performed MD simulations of liquid PC and formamide at the nanometer confinement. The confining slabs were modeled using crystalline solids with a unit cell corresponding to gold (0.4078 nm), with the (1,1,1) confining plane. The interactions between the gold atoms were calculated using the model presented in ref. ^29^, which provides accurate predictions of the densities and mechanical properties of gold as well as surface tension and interfacial properties of water–gold interfaces. The confined volume was filled with the molecular fluids and the number of molecules was adjusted to obtain the bulk density of the liquid (see below for values) in the middle of the film.

**Fig. 3:**
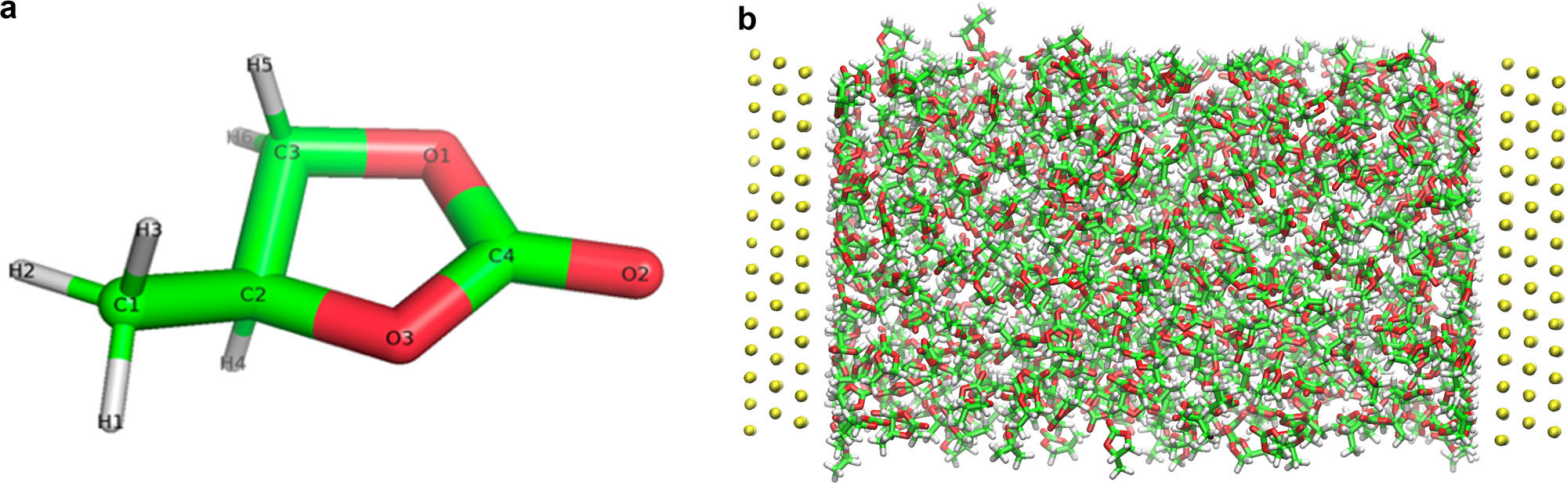
Illustrations of molecular dynamics simulations for PC. **a** Molecular structure of PC. **b** Representative configuration of PC confined between gold slabs (gray spheres). The distance between the gold layers in direct contact with PC is 6.5 nm. The oxygen, carbon, and hydrogen atoms in PC are colored in red, green, and white, respectively. The snapshot corresponds to the *y*–*z* plane, with the *z* vector being perpendicular to the gold slabs.

In Fig. 3, we show a representative snapshot of the confined system employed in our computations. The typical dimensions of our simulation box in the direction parallel to the slab were (*L*_x_, *L*_y_) = (4.03721, 3.49633) nm, and the slab–slab distance in the *z* direction was set to a set of selected values *L*_ss_ = (3.52, 6.52, 11.52, and 26.52) nm, in order to investigate the role of different confinement conditions.

PC was modeled using a fully atomistic forcefield derived initially with the Automated force field Topology Builder^30^. The simulations were carried out in the canonical (NVT) ensemble at 300 K and the trajectories involved long trajectories, typically 40 ns. Specific details on the simulation conditions and forcefield fitting are provided in Supplementary Note 2. The simulation of formamide was performed with a similar set up (the same slab–area and slab–slab separation), and an accurate forcefield that reproduces the dielectric constant, density, and surface tension of formamide.

**Fig. 4:**
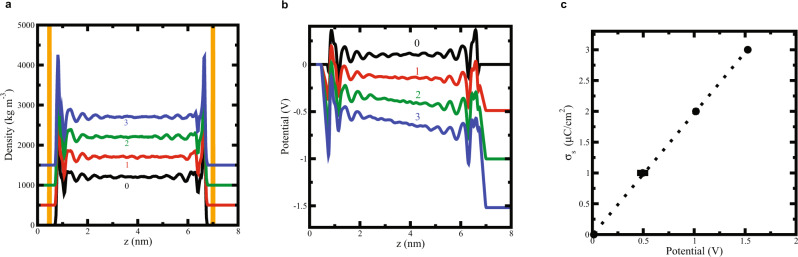
Results of molecular dynamics simulations for PC. **a** Density profiles of liquid PC at different charge densities, 0, 1, 2 and 3 µC cm^−2^ (The densities have been shifted vertically by 500 units for clarity). **b** Electrostatic potential drop for the same systems, as represented in the left panel; left and right electrodes are positively and negatively charged, respectively. **c** Variation of the voltage with the surface charge density. The straight line represents the linear fitting to the data. The computed capacitance for this system is 1.98 µF cm^−2^.

The capacitance was computed by confining the liquids between charged plates of opposite charge. We added the same charge to all gold atoms in the layer that is in direct contact with liquid. The capacitance density was calculated using: ${\widetilde{C}}_{{\rm{liquid}}}=d{\sigma }_{s}/{dV}$, where ${\sigma }_{s}$ is the surface charge and $V$ the electrostatic potential drop from the left to the right gold plates. Figure 4 shows typical density and electrostatic potential profiles. The walls induce strong layering in the liquid, and this results in significant changes in the electrostatic potential at the interfaces. We also show in Fig. 4 the dependence of the voltage with the surface charge. The data can be fitted very well to a linear regression, indicating that the capacitance of the confined film does not show a significant dependence on surface charge, or voltage. Similar results were obtained for formamide^31^.

The break in symmetry induced by the confining walls modifies the effective dielectric constant of the liquids. This is reflected in the capacitance density, which is significantly different from the value expected classically, namely, $\frac{\varepsilon {\varepsilon }_{0}}{d}$, where $\varepsilon$ and ${\varepsilon }_{0}$ represent the bulk dielectric constant and the vacuum permittivity, and *d* is the thickness of the liquids films. Using the bulk dielectric constant of PC we get capacitances of the order of 9 µF cm^−2^ for *d* = 6.5 nm. This capacitance is much higher than the one obtained in the simulations, 1.98 µF cm^−2^ (see Fig. 4—right panel), highlighting the important role than nanoconfinement plays in modifying the dielectric properties of the confined liquids.

The discrepancies observed here, between the predictions of simulation and of the macroscopic continuum theory, are in line with the findings of experimental and simulations studies of water under confinement^29,30^. The dielectric constant of water was found to only slowly converge toward to its bulk value, as the thickness of the nanofilms increased. The estimated dielectric constant of water confined between surfaces at 60 nm separation was only half of the bulk value^29^.

Comparing $\frac{\varepsilon {\varepsilon }_{0}}{d}$ and the simulated capacitance densities we found a significant reduction of the effective dielectric constant of the confined liquids for a slab-to-slab distance of 5 nm: for PC it is 11.7 in the nanogap vs. 66.4 in the bulk, and for formamide it is, correspondingly, 10.7 vs. 107.2. The capacitances for the confined slabs at 5 nm are: 2.07 µF cm^−2^ for PC and 1.90 µF cm^–2^ for formamide.

### Simulations of a capacitor with an ionic liquid electrolyte

We also computed the capacitance of ILs under confinement using the same approach as discussed above. We chose a widely used room temperature IL, 1-Butyl–3-methylimidazolium tetrafluoroborate (BMIM BF_4_). We employed a coarse-grained representation of the IL, using our previous studies of electrotunable lubrication^32,33^. These models reproduce well the thermodynamic, dynamic, and interfacial properties of the imidazolium BF_4_ ILs. The solid substrate was modeled using the parameters reported in^34^, an FCC lattice with parameter 0.36 nm, and with the (111) face in contact with the IL. The simulations were performed at 350 K to ensure faster dynamics. Unlike the molecular fluids, room temperature ILs screen the field induced by the charged plates, typically within about 2–3 nm from the surface. We simulated a sufficiently thick film to ensure the field is zero in the center of the nanofilm (see Supplementary Figure 2). The analysis of these simulations gave two potential drops one for the positive electrode and another one for the negative. We used these potential drops at different surface charge densities (0–3 µC cm^−2^) to obtain the small voltage (linear response) capacitances: 2.8 µF cm^−2^ for the positively charged electrode and 2.9 µF cm^−2^) for the negatively charged electrode. These values are of the order of those obtained before for room temperature ILs (see e.g., ^35^). The total capacitance, considering both interfaces is, 1.4 µF cm^−2^. This capacitance is similar to the one obtained with the molecular fluids investigated above.

Thus, the main advantage for using ILs for filling the nanogaps is not the elevated values of capacitance, but a relaxation of the requirement to keep the nanogap very small. Indeed, making the distance between the electrodes larger than the sum of the characteristic width of the double layers on the opposing electrodes will not affect the capacitance. On the other hand, ILs may also have advantages as nonvolatile lubricants.

### Calculating electrical current and power

The electrical circuit implementing the rotor device is illustrated in Fig. 1. The reservoir capacitor ${C}_{{\rm{fix}}}$ is initially disconnected from the rest of the system and momentarily connected to a DC voltage supply, for instance a battery, of voltage ${V}_{{\rm{ref}}}$. Therefore, the initial excess charge on ${C}_{{\rm{fix}}}$ is $q={C}_{{\rm{fix}}}{V}_{{\rm{ref}}}$. When the reservoir capacitor is connected to the rest of the circuit, i.e., to the rotor time-varying capacitors and to the electrical load, the excess charge is redistributed between the two rotor capacitors and reservoir capacitor but is overall conserved since the system is well isolated.

The governing equation for the charge, $Q\left(t\right)$, on the time-varying capacitor $C\left(t\right)$ for the electric circuit presented in Fig. 1 is given by (see Supplementary Note 4):

4 $$\frac{{dQ}}{{dt}}+\frac{Q\left(t\right)}{R}\left(\frac{1}{{C}_{{\rm{fix}}}}+\frac{1}{C\left(t\right)}\right)={j}_{0}$$

where ${j}_{0}$ is defined as:

5 $${j}_{0}=\frac{{V}_{{\rm{ref}}}}{R}$$

Since ${C}_{{\rm{fix}}}\gg C\left(t\right)$, Eq. (5) is approximated to:

6 $$\frac{{dQ}}{{dt}}+\frac{Q\left(t\right)}{{RC}\left(t\right)}=\frac{{V}_{{\rm{ref}}}}{R}$$

The general solution of the above equation can be written as (see Supplementary Note 4):

7 $$Q\left(t\right)=\frac{{V}_{{\rm{ref}}}}{{RI}\left(t\right)}\left(\int_{0}^{{t}}I\left({t}^{{\prime} }\right)d{t}^{{\prime} }+{RC}\left(0\right)\right),$$

where the integrand, $I\left(t\right)$, is given by:

8 $$I\left(t\right)={\rm{exp }}\left[\frac{1}{R} \int_{0}^{{t}}\frac{d{t}^{{\prime} }}{C\left(t^{\prime}\right)}\right]$$

To go further, specification of $C\left(t\right)$ is required. Introducing the following rescaling:

9 $$C\left(t\right)=N{C}_{{{\max }}}\,f\left(t\right)$$

We note that the function $f\left(t\right)$ must be continuous, periodic, and bounded between the ratio of capacitances:

10 $$\gamma \equiv \frac{{C}_{{{\min }}}}{{C}_{{{\max }}}},$$

and 1. Under these restrictions, we modeled $f\left(t\right)$ in the following way:

11 $$f\left(t\right)={{{\cos }}}^{2}\left(\frac{\omega t}{2}\right)+\gamma {{{\sin }}}^{2}\left(\frac{\omega t}{2}\right),$$

where $\omega$ is the angular velocity of the disc. Within this model, the general solution in Eq. (7) becomes (see Supplementary Note 5):

12 $$Q(t)= \, \exp \left[-\frac{2}{\omega \sqrt{\gamma }\tau }\arctan \left(\frac{{\left(\sqrt{\frac{1}{\gamma }}-1\right)\tan \left(\frac{\omega t}{2}\right)}}{1+\sqrt{\frac{1}{\gamma }}{\tan }^{2}(\frac{\omega t}{2})}\right)\right] \\ \, \times\{{V}_{{\rm{ref}}}\gamma {C}_{\max }\exp \left(-\frac{t}{\sqrt{\gamma }\tau }\right)+{j}_{0}\int_{0}^{t}\exp \left(-\frac{t-{t}^{\prime}}{\sqrt{\gamma }\tau }\right)\\ \, \exp \left[\frac{2}{\omega \sqrt{\gamma }\tau }\arctan \left(\frac{\left(\sqrt{\frac{1}{\gamma }}-1\right)\tan (\frac{\omega {t}^{\prime}}{2})}{1+\sqrt{\frac{1}{\gamma }}{\tan }^{2}(\frac{\omega {t}^{\prime}}{2})}\right)\right]d{t}^{\prime}\}$$

where $$\tau ={RNC}_{{\rm{max}}}$$.

From Eq. (12), we calculate both the electrical current:

13 $$j(t)=\frac{dQ}{dt}$$

and the power released on the electric load, averaged over a single period of current oscillations:

14 $${P}_{R}=\frac{\omega }{2\pi }{\int }_{0 }^{2\pi /\omega} R{j}^{2}\left(t\right){dt}.$$

### Angular velocity of the rotor

The disc’s angular velocity value, $\omega$, which is important for optimization of the rotor’s performance, is determined by the balance between generated power and dissipated power. Energy dissipates from the system mainly by two routes: viscous drag on the disc and electric work on the resistive load. As the amplitude of generated currents are diminishing with decrease of $\omega$ it is important to investigate the characteristic values for $\omega$ that can be reached with the device.

The external torque, ${\tau }_{{\rm{p}}},$ which generates the motion of the rotor under the variable pressing force, is roughly given by,

15 $${\tau }_{{\rm{p}}}\simeq r{F}_{{\rm{p}}}$$

where $r$ is the radius of the slide-crank disc and ${F}_{{\rm{p}}}$ is the force exerted on the shaft by the external force. To estimate ${F}_{{\rm{p}}}$, let us smear the loss of press momentum, $p$, over a period of pressure ${T}_{{\rm{p}}}$ (of the order of ~1 s) therefore replacing the time profile of ${F}_{{\rm{p}}}$ with the average force:

16 $${F}_{{\rm{p}}}\simeq \frac{p}{{T}_{{\rm{p}}}}$$

For example, in the case of foot press, the momentum loss can be roughly estimated as the mass of foot (~25 kg) times the velocity of press downwards (~1 ${\rm{m}}{{\rm{s}}}^{-1}$). The externally applied torque should be large enough to overcome the friction torque due to viscous drag on slide-crank disc. Considering a gap $l$ between the disc and the semi-circular electrode, the drag torque, ${\tau }_{{\rm{d}}},$ acting on all $N$ discs, is approximately given by (see Supplementary Note 3):

17 $${\tau }_{{\rm{d}}}\simeq \frac{1}{2}\pi \nu \omega {r}^{4}\frac{N}{l+\delta },$$

where $\nu$ is the dynamic viscosity of the lubricating fluid. The slippage length, $\delta ,$ is determined by corrugation of sliding energy surface at the interface between the fluid and the half-circular electrode. The slippage length strongly depends on the hydrophobic nature of the frictional interface and can reach a value between tens and hundreds of the liquid’s molecular diameter^36^. Clearly, in the case of molecular liquid with a gap of a few nanometers, the slippage length will be crucial for determining the drag torque. On the other hand, for an IL with a micron-sized gap, the effect of slippage length is negligible.

The overall balance of power in the system gives an estimate for the average rotational frequency under steady state conditions$:$

18 $${\tau }_{{\rm{p}}}\omega -{\tau }_{{\rm{d}}}\omega -{P}_{{\rm{R}}}=0$$

where ${P}_{{\rm{R}}}$ is the average power delivered on the load. Substituting the expression for ${P}_{{\rm{R}}}\left(\omega \right)$ given by Eq. (14) into Eq. (18) will give a transcendental equation on $\omega$. Before solving it, it will be instructive to simplify it in the limit of high viscous drag by neglecting ${P}_{{\rm{R}}}\left(\omega \right)$.

The validity of this approximation is analyzed in Supplementary Note 6 for the different setups of the rotor together with a full solution of Eq. (18). Within the made approximation, we can readily solve this equation for $\omega$; using Eqs. (15)–(17) the solution reads:

19 $$\omega =\frac{2{p}_{{\rm{f}}}\left(l+\delta \right)}{\pi \nu {T}_{{\rm{p}}}{r}^{3}N}.$$

For example, in the case of a periodic foot press, PC as lubricating liquid and given the following typical values of system parameters: ${p}_{{\rm{f}}}=25{\rm{Ns}}$, $\nu =2.5{\rm{mPas}},$ $N={10}^{2},$ $r={10}^{-2}{\rm{m}},\delta =50{\rm{nm}},{\rm{l}}=5{\rm{nm}},{T}_{{\rm{p}}}=1{\rm{s}}$ we get, $\omega \approx 3.5{{\rm{s}}}^{-1}.$ Note that, in practice, the input shaft would be connected to a gear set and a freewheel mechanism (as present on the rear wheel hub of a bicycle) in order to enable the frequency of harvester’s shaft to exceed the frequency of the foot press. Furthermore, a flywheel with an optimized second moment of inertia would be used to maintain shaft rotation between intermittent foot presses. These basic mechanical components are outside the scope of this proof of concept study. For ILs, the gap size can be chosen much larger, on the scale of micrometers, which reduces drag but, on the other hand, the viscosity of ILs is much higher, which would increase the drag. However, it is possible to dramatically decrease the viscosity of ILs, without significantly changing their electric screening properties, if we consider a mixture of IL with small amounts of acetonitrile (up to 16 wt%). Such a mixture can reach lower viscosities as small as $5{\rm{mPas}}$^37^. In the case of a foot press with IL mixture and system parameters, ${p}_{{\rm{f}}}=25{\rm{Ns}}$, $\nu =5{\rm{mPas}},$ $N={10}^{3},$ *r* = 10^−2^ m, *l* = 10 μm*,T*_p_ = 1 s, we get $\omega \approx 32{{\rm{s}}}^{-1}$.

### Steady state behavior at large angular velocities

The time regime at which steady state is achieved is given by $t\gg \sqrt{\gamma }\tau$. At this regime, the device forgets about the initial conditions and remains at a steady and periodic state.

At the stationary regime, for large enough angular velocities:

20 $$\omega \gg \frac{1}{\sqrt{\gamma }N{C}_{{{\max }}}R},$$

the charge on the time-varying capacitor, as given by Eq. (12), will oscillate around the constant value ${Q}_{1}=\sqrt{\gamma }{V}_{{\rm{ref}}}N{C}_{{\rm{max }}}$. These oscillations will have a steady state amplitude proportional to $\frac{1}{\omega }$ (see Supplementary Note 5). For such high frequencies, the electric current, ${j}_{{\rm{ss}}}\left(t\right)$, at steady state, is given by (see Supplementary Note 5):

21 $${j}_{{\rm{ss}}}\left(t\right)={j}_{0}\left(1-\frac{2\sqrt{\gamma }}{1+\gamma -\left(1-\gamma \right){{\cos }}\left(\omega t\right)}\right).$$

The average of this current over a period is independent of the angular frequency. Indeed, while the amplitude of oscillations in the charge of the rotor drops like $\frac{1}{\omega }$, their frequency increases with $\omega$ so that these two effects fully compensate each other in this regime. The average power on the load in this regime is also independent of frequency value, and is acquired by substituting Eq. (21) into Eq. (14), which gives:

22 $${P}_{0}=\frac{{V}_{{\rm{ref}}}^{2}}{R}\frac{{\left(1-\sqrt{\gamma }\right)}^{2}}{2\sqrt{\gamma }}$$

This result remains true irrespective of the liquid setup used. An interesting and immediate conclusion comes out from Eq. (22): the average power at high frequencies is independent of the extremal capacitance values, ${C}_{{\rm{max }}}$ and ${C}_{{\rm{min }}}$, it depends only on the ratio, $\gamma$ (Eq. (10)). However, since this high frequency regime is defined by angular velocities satisfying the criteria in Eq. (20), increasing the maximum capacitance ${C}_{{\rm{max }}}$ will enable us to reach this plateau power at smaller frequencies.

A similar tradeoff exists for $\gamma$; as $\gamma$ decreases, ${P}_{0}$ increases roughly like $\frac{1}{\sqrt{\gamma }}$. We can lower $\gamma$ effectively indefinitely by increasing the thickness of the low dielectric layer, however as $\gamma$ decreases, the plateau power is reached at a higher angular velocity and vice versa. Information provided by Eq. (22) with its criterion, Eq. (20), is central for the optimal design and operation of the device.

### Heat management

Another point to consider is thermal balance. The rotation of the whole rotor will generate heat due to the drag created by the lubricating liquid. Subsequently, this heat will result in an increase in temperature of the device. Various heat dissipation mechanisms counteract heat generation toward a steady state working temperature. Overheating of the device may cause various problems, and so, it is important to account for thermal balance and increase in temperature due to rotor rotation. The rate of heat generated due to drag is approximately given by:

23 $$\frac{d{Q}_{{\rm{in}}}}{{dt}}={\tau }_{{\rm{d}}}\omega$$

While the rate of heat evacuated from the device, dominantly by conduction, is approximately given by:

24 $$\frac{d{Q}_{{\rm{out}}}}{{dt}}=-\frac{\kappa_T S}{L}\left(T\left(t\right)-{T}_{{\rm{env}}}\right),$$

in a simple 1D heat conduction approximation, where ${T}_{{\rm{env}}}$ is the environmental temperature, $T\left(t\right)$ is the device temperature, $L$ is the thickness of the bottom layer which separates the rotor from the environment ($L \sim \frac{1}{2}{\rm{cm}}$), $S$ is the area of that layer which depends on the number of stages, and $\kappa_T$ is the thermal conductivity of the separating layer (for regular rubber $\kappa_T \approx 0.14{\rm{W}}/{\rm{m}}{\rm{K}}$).

The balance between heat generation and evacuation will give an estimate for the steady state temperature of the device, ${T}_{{\rm{ss}}}$:

25 $${T}_{{\rm{ss}}}-{T}_{{\rm{env}}}=\frac{{\tau }_{{\rm{d}}}\omega L}{\kappa_T S}$$

For a 1 cm long device, and 1 cm disk radius, we get $S=2\times {10}^{-4}{{\rm{m}}^2}$, and an increase in temperature of about 0.15 K in the case PC liquid with of ${10}^{2}$ stages and an increase in temperature of about 2.8 K in the case of IL with ${10}^{3}$ stages. Overall, for both cases the temperature increase is quite small and therefore this device may readily be implemented in shoes and worn with comfort.

### Limitations on priming voltage

Finally, we investigate the possible values for the priming voltage, ${V}_{{\rm{ref}}}$, used to momentarily charge the fixed capacitor ${C}_{{\rm{fix}}}$. On the one hand, a higher priming voltage will dramatically increase the power output we get from the device, however, the priming voltage is limited by the safety voltage, which depends on the specific implementation of the rotor, and by the dielectric breakdown voltage for the lubricating liquid. Usually, 50 V DC is taken as a safety cap voltage for such devices. The cap voltage $V$ for dielectric breakdown in molecular liquids is determined by the ionization energy of the lubricating liquid ${W}_{{\rm{ion}}}$, and is approximately given by:

26 $$V\approx \frac{l}{s}\frac{{W}_{{\rm{ion}}}}{e}$$

where $s$ is the characteristic length of a molecule in the liquid and $e$ is the electron charge. The ionization energy for both formamide and PC is about 10 eV. Therefore, for a gap of $5{\rm{nm}}$, the cap voltage due to electrical breakdown is about 170 V, which is well beyond the safety cap. For ILs, voltage is limited by the so called electrochemical window: the range of electrode potentials within which electrochemical reactions involving IL ions does not take place neither at the anode, nor at the cathode. Within that range there will no spontaneous capacitor discharge. Usually, in ILs, if the potential drop between the bulk and each electrode do not exceed 2V, such reactions will not yet take place. We will therefore consider a maximum of 4 V priming voltage for ILs.

### Numerical performance estimates

Let us evaluate the output of the rotor device, in terms of electric current and power through the electric load $R$, presenting the results for two types of liquid setups: (1) ${10}^{2}$ stages with PC lubricant in nanogaps of $l=5{\rm{nm}}$, and, (2) ${10}^{3}$ stages with ILs mixture lubricant in wide gaps *l* = 10 μm. The discs are the same on both setups, i.e., 1 cm radius and dielectric coatings ${d}_{{\rm{L}}}=200{\rm{nm}}$, ${d}_{{\rm{H}}}=5{\rm{nm}}$ as discussed in the theory section. It should be noted that while the two setups are different, both in number of stages and gap size, they can be compared by considering their response to the same generative power of press for both setups. We generally considered more stages for IL setup since the gap in this setup is wider and the friction is lower, thereby giving a larger response in terms of angular velocity for the same external force. The value of the maximum capacitance per area of a single stage, was calculated using Eq. (2) by substituting in the relevant lubricant capacitance density ${\widetilde{C}}_{{\rm{liquid}}}$ acquired from the simulations. For IL mixture setup we got $\frac{{C}_{{\rm{max }}}}{A}=1.35$ µF cm^−2^ per stage and for PC setup we got $\frac{{C}_{{\rm{max }}}}{A}=1.88$ µF cm^−2^ per stage.

**Fig. 5:**
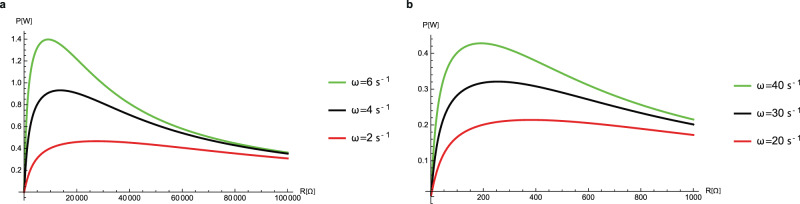
The average power profile against the electric load for different rotation frequencies. Curves are plotted for two different lubricating liquids, **a** PC and **b** IL mixture. Each case is presented for three different rotation frequencies (typical and different for each case, due to the difference in drag torques). Parameters: **a** $N={10}^{2}$, ${V}_{{\rm{ref}}}=50{\rm{V}}$; **b** $N={10}^{3},{V}_{{\rm{ref}}}=4{\rm{V}}$. Note that the optimal power regimes and structures for these two systems (**a** and **b**) are achieved for different number of stages, frequencies and voltages; and the graphs of the power, in **a** and **b**, have been plotted for different parameter domains.

**Fig. 6:**
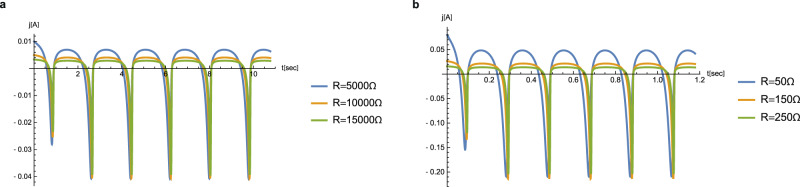
Temporal profile of the electric current. Curves are plotted for two different lubricating liquids, **a** PC and **b** IL, each presented for three different electric loads. Parameters: **a** $N={10}^{2},\omega =3.5{{\rm{s}}}^{-1}$, ${V}_{{\rm{ref}}}=50{\rm{V}}$; **b** $N={10}^{3},\omega =32{s}^{-1},{V}_{{\rm{ref}}}=4{\rm{V}}$.

**Fig. 7:**
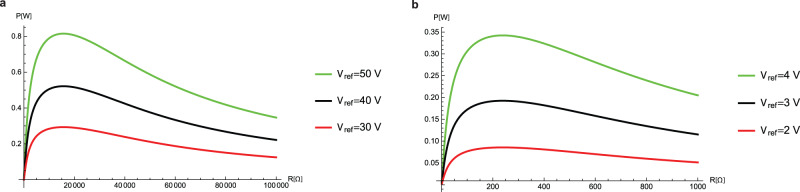
The average power profile against the electric load for different priming voltages. Curves are plotted for two different lubricating liquids: **a** PC and **b** IL, each presented for three different priming voltages. Parameters used: **a** $N={10}^{2}$, $\omega =3.5{{\rm{s}}}^{-1}$; **b** $N={10}^{3},\omega =32{{\rm{s}}}^{-1}$.

The steady state average power was calculated using Eq. (14) and plotted vs. the electric load for different frequencies in Fig. 5. As discussed above in the theory section, and as seen in Fig. 5, an optimum load exists for maximum power. The optimal electric load is very different for PC and IL, mainly because of their different operational angular frequencies. The current as a function of time for different electrical loads, in the vicinity of the optimal load, was calculated using Eq. (13) and is presented in Fig. 6. The current profile stabilizes after a short time interval, and afterwards persists in a periodic fashion. A power vs. load profile for different priming voltages is shown in Fig. 7.

**Fig. 8:**
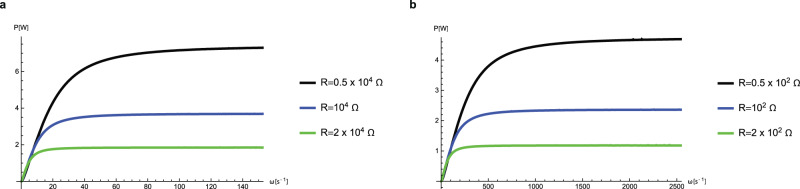
Average power profile as a function of rotational frequency for different loads. Curves are plotted for: **a** PC with $N={10}^{2}$ stages and priming voltage ${V}_{{\rm{ref}}}=50{\rm{V}}$; **b** IL with $N={10}^{3}$ stages and priming voltage ${V}_{{\rm{ref}}}=4{\rm{V}}$. And, again, the parameter domains are different for **a** and **b**; the arguments for this are explained in the caption of Fig. 6.

Finally, the power on a given load $R$, as a function of a wide range of rotational frequencies, $\omega$, as calculated from Eq. (14), is shown in Fig. 8.

It shows that, for very large frequencies of rotation, the power on the load becomes independent of the angular velocity just as we predict in the theory section. Each curve reaches the plateau of power at a different frequency; as the load, *R*, increases, the transition frequency, ${\left(\sqrt{\gamma }N{C}_{{\rm{max }}}R\right)}^{-1}$, decreases as in the discussed case of increasing ${C}_{{\rm{max }}}$. Similarly, the transition frequency gets smaller with increase of $N$. The value for the power on the plateau, described by Eq. (22), perfectly matches the numerical result presented in Fig. 8, for both IL and molecular liquid setups, for all resistances and priming voltages.

The estimated power density of our proposed device is higher than that of previously reported designs because of the large maximum capacitance. For example, data for an RED^6^ with a capacitance of 16 ${\rm{nFc}}{{\rm{m}}}^{-2}$ can yield energy of up to 38 μJ cm^−2^ per cycle at 63 ${\rm{V}}$. For comparison, the left-hand plot in Fig. 8 indicates an expected output power of 1 W for ${\omega }=5{{\rm{s}}}^{-1}$ at 50 ${\rm{V}}$ bias for 100 stages, and this corresponds to an energy yield of $\sim 4$ ${\rm{mJc}}{{\rm{m}}}^{-2}$ per stage per revolution.

## Discussion

The suggested disc-electrode gap of 5 nm for molecular lubricants and 10 μm for IL operation raises challenges for the practical implementation of the proposed energy harvester. However, such thin gaps may be achieved in practice through passive control approaches. Lubricating oil films with thicknesses ranging from nm to μm already occur naturally in ubiquitous components such as bearings and seals^38^. In these components, the integral of the normal pressure between sliding surfaces must be in equilibrium with the applied load and this is achieved through regulation of the film thickness. For a given applied load, the value of equilibrium film thickness depends on lubricant properties, sliding speed and contact geometry, and can therefore be controlled by adjusting these parameters. The contact geometry may be modified by using standard microfabrication methods to introduce texture to the sliding surfaces^39^. This widely used approach may be implemented in the design of the capacitance energy harvester in order to ensure the required nm/μm-scale gap.

In order for the self-regulation mechanism to work, the discs and electrodes will need to be free to make slight tilt adjustments to compensate for parallelism errors, while remaining coupled to the rotor shaft or side support for transmission of the drive torque. This problem has been addressed in earlier work on mm-scale MEMS pad bearings^40^. The preferred solution for the present device would be to locally thin these parts to make them compliant in the tilt axes while maintaining sufficient torsional stiffness in the rotation axis.

It should be noted that the suggested disc-electrode gap distances (5 nm for molecular liquid setup and 10 µm for IL setup) are viable in terms of lubrication performance. This is supported by friction measurements and simulations demonstrating that confined molecular and ILs remain fluid down to a film thickness of few molecular layers, which corresponds to the surface separation of 5–10 nm^33,34,41–44^. Importantly, these nanoconfined liquid layers exhibit ultralow friction coefficients of 0.001–0.05^33,42–45^, thus providing a very low energy dissipation between the surfaces in relative motion. This is the crucial condition for the effective and durable operation of the proposed device.

For any system with a liquid lubricant, loss of the lubricant through evaporation must be minimized if long-term operation is required. In this work we intentionally chose PC for its low volatility at room temperature and atmospheric pressure. This is evident from its extremely low vapor pressure which has been measured^46^ to be only 3 Pa at 298 K. room temperature ILs of the kind proposed here also exhibit very low vapor pressures. Furthermore, in confined nanometer films, strong interaction with substrate further reduces volatility of molecular and ILs. Finally, evaporative loss can be further reduced by encapsulating the rotor in a sealed package; in this case lubricant evaporation could occur only via the rotary seal where the axle passes through the chamber wall.

The possible power densities that can be achieved with both setups, using either molecular or IL, are similar (see Fig. 8). However, from a practical point of view, the molecular liquid setup requires fabrication of devices with nanometer-sized gaps, which could be complicated. Whereas for the IL setup, one can choose much larger gaps, of micron size, which are more readily fabricated. On the other hand, ILs are more expensive and may present higher viscosity in the bulk, although, between polarized electrodes the effects of electrotuneable friction could still make them very good lubricants^45,47–50^.

In this paper we give estimates for the performance of the device within specific design details in the framework of a shoe sole implementation (a small-scale application). The question may, however, be raised, how minute changes to the liquid’s gap thickness would affect the power output of the device, provided we keep the total number of stages constant. For the IL lubricant setup, provided the gap’s width remains much larger than the narrow, nanometer sized width of the electrical double layer, the maximum capacitance of the rotor will not change due to such fluctuations. Variation in the drag-force will neither be significant as long as the changes in the gap widths are of the order of tenths of a micro-meter. Therefore, we can safely assume that the overall power output of the device will remain constant under minute variations of the liquid’s gap widths. For the molecular liquid lubricant setup, in order to give a fair estimate as of how the power output changes with gap size, we will first need to know how the capacitance of PC molecular liquid changes with the gap width. That’s not a simple task, and the major instructions about it should be obtained from specially performed experiments (for an example of such studies performed on water see^28^ Fumagali et. al). In the absence of such experiments for PC, we have evaluated that effect by MD simulations, as described in the paper. In order to give a realistic estimate for the capacitance of PC in our nanogap structures we performed MD simulations for a single gap size of 5 nm, but we did not pursue this further for different gap sizes. Instead, we give a qualitative answer as to how the power output changes with gap size for molecular liquids. The dielectric capacitance of the liquid will change, approximately, in inverse proportion to the gap size, and while the liquid’s drag will reduce as the gap size increases, that latter effect will be negligible, provided the gap size is still much smaller than the slip-length. We therefore qualitatively estimate that even if we increase the gap size by two folds, i.e., to 10 nm, the power output of the device will drop down, but will still remain significant, of the order of 1 W.

We comment that the fixed capacitor ${C}_{{\rm{fix}}}$ can be swapped with another time-varying capacitor. Such a setup may produce more output power, however in order for such a device to work with high efficiency, the two time-varying capacitors would have to be in antiphase at all times. Such a restriction might lead to additional design complications.

Correct operation of the proposed device requires that the disc and pad surfaces are smooth down to nanoscale level and free from asperities that prevent the desired gap from being achieved. However, chemical mechanical polishing techniques that can produce such smooth surfaces over large areas are well-known from the semiconductor industry. It is also the case that metal coatings, such as sputtered chromium, are sufficiently smooth and durable so as to enable the conductive electrode to be produced while maintaining sub-nanometer roughness. Furthermore, the production of a micro-thrust pad bearing device able to maintain an uninterrupted nanometer gap between silicon discs of 2 mm diameter is something that has been successfully demonstrated^40^.

Huang et al.^23^ have recently suggested a superlubric nanogenerator (SLNG) device based on a time-varying capacitor at a frictional solid–solid interface, in which superlubricity prevents extensive wear and energy dissipation. In our device, the matter filling the gaps between electrode and dielectric is liquid, whereas in the SLNG there is a nanometer-sized solid dielectric made of silicon-dioxide. While both devices are based on time-varying capacitance, they differ first of all in the mechanism of operation. The suggested SLNG produces a change in capacitance by sliding one electrode tangentially across the nanometer-sized solid dielectric (situated between the two electrodes), thereby changing the capacitor’s effective contact area. Whereas in our device a variation of capacitance is induced by changing the dielectric structure of each capacitor upon rotation of the rotor device. It should be mentioned that the fabrication of a dielectric layer of silicon-dioxide with an atomically flat layer of nanometer thickness and macroscopic lateral dimensions, is a very difficult task. In our suggested device we offer the possibility to choose between micro-meter gaps filled with IL and nanometer gaps filled with molecular liquid, which, eventually, give the same order of magnitude of the generated power as the SLNG device. Precise comparison of the power densities is, however, difficult since Huang et al.^23^ did not study in their paper the momentum source and momentum transfer mechanism. In our paper we considered an example of a momentum source corresponding to the pressing of a shoe, and a momentum transfer mechanism from which we got the rotational frequency of operation and the predicted power density, which results in approximately $\sim 4{\rm{mjc}}{{\rm{m}}}^{-2}$ per stage per revolution (see the left-handed plot in Fig. 8). Secondly, the IL-lubricant version of our device is a low-voltage device and it does not require small nanogaps, as such it is good for lubrication, thermal control, and device robustness. It can give very good results for small voltages, below 4 V, which makes it handy to use in portable devices. The dielectric-liquid version will demand smaller nanogaps and higher voltages, but it will be cheaper as it can use standard solvents, like PC.

The principle of the proposed device in this paper can be used in upscaled applications. Utilization of wind or water flow in upscaled portable or domestic applications will change the input external pressures, viscous drags and the maximal value of capacitance as the area of the electrode plates can be made larger. A large system composed of many rotor units will dissipate heat differently depending on the topology in which rotors were stacked together. So long that we increase the size of the system by stacking rotor units from the sides, like in a “2D sheet”, then the negligible temperature increase that we calculated in Eq. (25) would approximately not change. The addition of units from the sides comes hand-in-hand with a corresponding increase in the lateral surface area, from which heat is dissipated. Thus, each rotor creates and dissipates heat approximately independently of all other units. If, on the other hand, rotors are stacked on top of one another, then in order to keep a negligible temperature increase, a heat management solution must be introduced, like heat sinks in between consecutive layers. Further analysis of the functioning of such upscaled devices could be performed along the lines discussed above. We are not presenting it here but all concepts for doing that are well defined.

In conclusion, the idea of a rotary variable capacitor dates back to the 1890s when the Hungarian engineer Dezső Korda was granted a patent for a device in which the capacitance was adjusted by varying the overlap between two interleaved stacks of semi-circular plates, one of which could be rotated while the other remained fixed. Korda’s device formed the tuning element in virtually all radio receivers until the 1970s when digital frequency synthesis started to take over; variable overlap rotary capacitors have also been proposed for use in electrostatic machines for high-voltage DC power generation^51,52^ and for small-scale energy harvesting^53^. In this work we have investigated an alternative rotary generator design in which the thickness and permittivity of the dielectric vary with the rotor position rather than the plate overlap area.

We have explored a “shish-kebab” design of capacitive rotor at a scale suitable for current generation from walking; the same design could be upscaled for less compact applications. Whereas as discussed in the Introduction, the idea of capacitive current generation is itself well-known, the scenarios considered and mathematically described in detail in this paper are new. The theoretical analysis revealed a number of findings and several nontrivial lessons:

1. We have found that, in the stationary regime and at large frequencies of rotation, the electrical power generated saturates to a maximal level, and this power does not depend on the extremal values of the variable capacitance, but only on the ratio of the maximal to minimal values. We have derived a simple, but non-trivial formula that describes this regime.
2. The approach to this regime, however, depends on the maximal value of capacitance. The larger maximal capacitance, the lower the frequency at which the maximum power is reached. Thus, operating at intermediate frequencies, it will, of course, be beneficial to maximize the capacitance.
3. We have shown that the device can be operated with both molecular liquids and ILs as lubricants between the rotating and stationary plates and compared features and differences in using these two options.
4. We have investigated how the generated electrical power depends on the applied electric load and established the range of the loads providing the maximal power, depending on the rotational frequency.

The paper rationalizes the physical meaning of all these findings.

The estimates of the possible volumetric or gravimetric power density come out promising for portable applications. It is too early to discuss how practical and economic these devices will be, before conducting experimental tests of the proposed scenarios and building the first laboratory prototypes based on them. However, the principle and details of operation of the proposed class of devices have been made clear in the reported work. While the component parts of the proposed device are not expensive, a significant initial investment is likely to be required to develop a suitable manufacturing process. However, if this is done correctly then it should be possible to achieve low unit costs in large scale production. Given the market potential it would certainly be worthwhile to proceed along these lines, and the theoretical analysis presented here will provide guidelines for this work. If successful, there will be a lot of room for smart engineering of practical capacitive rotor devices for various sustainable energy applications.

## Supplementary information

Supplementary Information

## Data Availability

All data are available within the article and Supplementary Information.
