# Addendum: Theoretical demonstration of a capacitive rotor for generation of alternating current from mechanical motion

**DOI:** 10.1038/s41467-023-35901-w

**Published:** 2023-01-30

**Authors:** Ehud Haimov, Aidan Chapman, Fernando Bresme, Andrew S. Holmes, Tom Reddyhoff, Michael Urbakh, Alexei A. Kornyshev

**Affiliations:** 1 School of Physics and Astronomy, Raymond and Beverly Sackler Faculty of Exact Sciences, Tel-Aviv University, Tel-Aviv, Israel; 2 Department of Chemistry, Faculty of Natural Sciences, Imperial College London, Molecular Sciences Research Hub, White City Campus, London, UK; 3 Thomas Young Centre for Theory and Simulation of Materials, Imperial College London, South Kensington Campus, London, UK; 4 Department of Electrical and Electronic Engineering, Faculty of Engineering, Imperial College London, South Kensington Campus, London, UK; 5 Department of Mechanical Engineering, Faculty of Engineering, Imperial College London, South Kensington Campus, London, UK; 6 School of Chemistry, Raymond and Beverly Sackler Faculty of Exact Sciences, Tel-Aviv University, Tel-Aviv, Israel

**Keywords:** Devices for energy harvesting, Electrical and electronic engineering, Mechanical engineering, Electrochemistry, Electronics, photonics and device physics

Addendum to: *Nature Communications* 10.1038/s41467-021-23891-6, published online 16 June 2021

The paper proposed a new mechanism for alternating electrical current generation by a rotor system. The rotor comprises many repeating capacitive units which, upon rotation, vary their capacitance coherently between a maximum and a minimum value. The periodic changes in the total capacitance at a controlled, given voltage generate an alternating current in the circuit.

As depicted in figure 2 in the original article, each capacitance unit in the rotor comprised a stationary half-circular metal electrode and a rotating pedal with some gap in between the two. The entire system was drenched in an operating liquid that filled the gaps and performed, simultaneously, as both a capacitance enhancer and a lubricant. In the paper we suggested two different kinds of operating liquids: dielectric liquid and ionic liquid. In the paper we stated that the only difference in design when using the different kinds of liquids would be the gap size and the number of repeating units. However, recently we realized that confinement of ionic liquids between surfaces at large separations (much larger than nm ones) would prevent the rotor from reaching the predicted performance. The spreading of ionic liquid over a large gap between the metallic electrodes leads to their capacitive coupling thus making negligible capacitance changes as the rotor rotates.

This shortcoming in the design of the system with ionic liquids can be corrected immediately by introducing minor modifications to our original set-up, depicted in Figure 1 of the addendum. Essentially there are three modifications: covering the metallic parts of the pedal (and axle) by a dielectric coating to prevent a connection between the liquid and the metal, thickening or shifting the low dielectric coating so that it entirely fills the gap as it approaches the semi-circular metal electrode, and, keeping only one stationary electrode between each two adjacent pedals. In this slightly modified design, the additional low dielectric coating will force ionic liquid out of the gap as it rotates, thus disconnecting the stationary and rotating electrodes. It should be noted that the new design can be realized without changing the minimum nor maximum capacitance of the rotor.

**Fig. 1:**
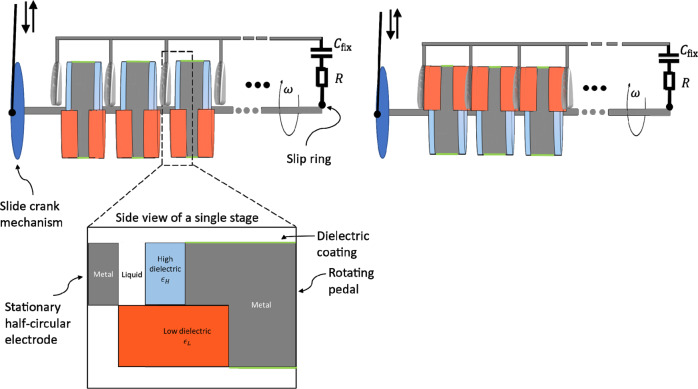
Modified rotor design for operation in ionic liquid. Left and right panels show the rotor at different rotational phases, that of maximum and that of minimum capacitance correspondingly. Stationary electrodes are designed to have edge thickness of a few orders of magnitude thinner than their faces, or/and they can be covered by thick low polar dielectric film. As such they will not contribute noticeable capacitance.

It should be stressed that the original design does not need any correction for the operation of the rotor in a dielectric fluid. Operating with the original design in an ionic liquid environment must generate negligible currents; observing this will be an additional proof of the principle presented in the paper.

In the new design proposed for the ionic liquid case, to make sure that large variation of capacitance is achieved, the edges of the stationary half-disk electrodes must be orders of magnitude thinner than the half-disk radii; alternatively, they may be covered by thick dielectric film so that the contribution to capacitance from the edges will be negligible.

It is essential to note that all elements of the theory, as well as the computer simulations described in the paper and figures shown in the results section are correct. The slight change in the design of the rotor in the case of ionic liquids may result in a different speed of rotation than that of the original design, however, this was already considered in the paper by showing power profiles for different rotational speeds (see Figure 5 in the original article).

