# Supplementary material for: Theoretical demonstration of a capacitive rotor for generation of alternating current from mechanical motion
Source: Nat Commun. 2021 Jun 16;12:3678. doi: 10.1038/s41467-021-23891-6 (PMC8209174; doi:10.1038/s41467-021-23891-6)
Supplement: Supplementary file 1 — Supplementary Information [file 41467_2021_23891_MOESM1_ESM.pdf]

Supplementary information for the paper:

THEORETICAL DEMONSTRATION OF A CAPACITIVE ROTOR FOR GENERATION OF ALTERNATING CURRENT FROM MECHANICAL MOTION

E. Haimov, A. Chapman, F. Bresme, A. Holmes, T. Reddyhoff, M. Urbakh, and A. A. Kornyshev

Supplementary Note 1: Capacitance Calculations

Consider the system shown in Supplementary Figure 1.

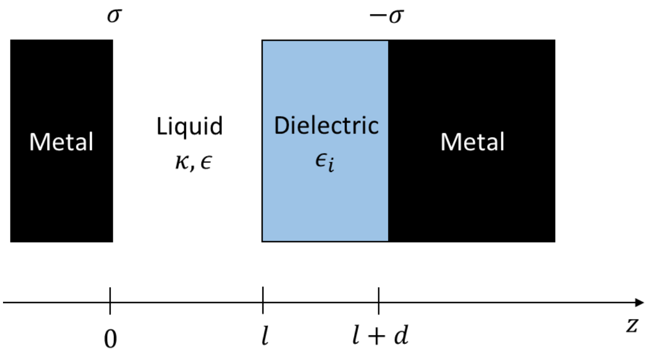

Supplementary Figure 1: Single capacitor made of a stationary electrode and a rotating pedal which contains both a second electrode and a dielectric ϵ_i connected to it. In between the first electrode and the dielectric is an electrolyte solution of Debye length κ^{-1} and dielectric constant of ϵ.

The equation of motion for the electric potential $\phi(z)$ is given by the linearized Poisson-Boltzmann equation (Debye-Huckel):

$$\frac{d^2\phi}{dz^2} = \kappa^2(\phi - \phi_0) \quad (1)$$

The electric potential everywhere is then given by,

$$\phi(0 \le z \le l) = Ae^{-\kappa z} + Be^{\kappa z} + \phi_0 \quad (2)$$

$$\phi(0 \le z \le l) = Ae^{-\kappa z} + Be^{\kappa z} + \phi_0 \quad (3)$$

$$\phi(a \le z \le l + d) = Dz + E \quad (4)$$

$$\phi(z \ge l + d) = const. \quad (5)$$

The boundary conditions are:

- Potential at $z = 0$:

$$A + B + \phi_0 = U \tag{6}$$

- Continuity of potential at $z = l$:

$$Ae^{-\kappa l} + Be^{\kappa l} + \phi_0 = Dl + E \tag{7}$$

- Continuity of displacement field at $z = l$:

$$\epsilon\kappa(Be^{\kappa l} - Ae^{-\kappa l}) = \epsilon_i D \tag{8}$$

- Potential at $z = l + d$:

$$D(l + d) + E = 0 \tag{9}$$

In addition to the boundary conditions, the system is overall electroneutral, i.e.,

$$\int_0^l \rho(z)\, dz = 0 \tag{10}$$

where,

$$\rho(z) = -\frac{2e^2 c_0}{k_B T}(\phi(z) - \phi_0) \tag{11}$$

which gives the relation:

$$B = A\frac{1 - e^{-\kappa l}}{1 - e^{\kappa l}} \tag{12}$$

By solving Supplementary Equations (6) - (9) and (12) for the parameter A, we get:

$$A = U\frac{\epsilon_i}{2\epsilon_i(1 - e^{-\kappa l}) + \epsilon\kappa d(1 + e^{-\kappa l})} \tag{13}$$

The capacitance per unit surface is given by:

$$C_S = \frac{\sigma(z = 0)}{U} = \frac{-\frac{\epsilon}{4\pi}\frac{d\phi}{dz}\Big|_{z=0}}{U} = \frac{\epsilon\kappa}{4\pi}\left(\frac{A}{U} - \frac{B}{U}\right) \tag{14}$$

Plugging in Supplementary Equations (6) and (14), we get:

$$C_S = \left(\frac{8\pi}{\epsilon\kappa}\tanh\left(\frac{\kappa l}{2}\right) + \frac{4\pi d}{\epsilon_i}\right)^{-1} \quad (15)$$

Supplementary Note 2: Molecular Simulations

We performed long canonical (NVT) simulations, 60ns, of the liquids to compute dielectric constants, and NPT simulations to calculate bulk densities. We employed the v-rescale [1] thermostat to set the system temperature and the Berendsen barostat [2] for the constant pressure simulations. The slab simulations, as indicated in the main text, involved typically 40 ns trajectories. We employed the 3D correction [3] to compute the electrostatic interactions using the Ewald summation method, and the Lennard-Jones interactions were truncated at 1 nm using a spherical cut-off. The cross interactions were computed using geometric combination rules.

The system was surrounded by a large vacuum gap in the z direction, to prevent image-image interactions. The total length of the simulation in this direction, L_z, was $3L_s$, at least. The simulations of the slab systems consisted of ~500 PC or ~1000 formamide molecules. The gold slabs consisted of 588 atoms. 196 atoms in the layer next to the PC liquid were assigned electrostatic charges to perform the capacitance computations. The position of the gold slabs was restrained to prevent drift and maintain the desired level of confinement. All the trajectories were integrated with the Leap Frog algorithm, a timestep of 2fs, and the code GROMACS 2020.

The initial geometry for PC was obtained from ATB following optimization with B3LYP/6-31G*, with the initial charges estimated using the ESP method of Merz-Kollman [4,5] Intermolecular and intramolecular parameters with parameters were taken initially from the GROMOS 54A7 forcefield. PC is a chiral molecule. All the simulations presented here were performed with the S-enantiomer. We performed control simulations using the R enantiomer obtaining the same results for density and dielectric constant, within the statistical uncertainty of our computations.

The initial forcefield for PC over-predicted the density of the liquid at 300 K, 1295.1 ± 1.1 kg/m^3 (1197.6 kg/m^3) and the dielectric constant 73.4 ± 3.5 (Exp. 64.7). To improve the accuracy of the model we modified the Lennard-Jones parameters using the Amber94 model and the partial charges [6,7]. Supplementary Table 1 contains the final optimized parameters.

With these changes the predicted average density and dielectric constant are 1201.1 ± 0.4 kg/m^3 and 66.4 ± 3.6, in good agreement with the experimental results.

Atom	Charge (e)	σ (nm)	ε (kJ/mol)
O1	-0.4141	0.3167	0.8786
O2	-0.5632	0.3167	0.8786
O3	-0.4147	0.3167	0.8786
C1	-0.1133	0.3638	0.4577
C2	0.1141	0.3638	0.4577
C3	0.0979	0.3638	0.3598
C4	0.8651	0.3638	0.3598
H1	0.0672	0.2835	0.0657
H2	0.0532	0.2835	0.0657
H3	0.0579	0.2835	0.0657
H4	0.0877	0.2835	0.0657
H5	0.0816	0.2835	0.0657
H6	0.0806	0.2835	0.0657

Supplementary Table 1: Charges, effective diameters and interaction strengths employed in the simulations of PC performed in this work.

Simulations of a capacitor with Room Temperature Ionic Liquids

The capacitances were obtained by analyzing trajectories spanning 16 ns, following the same procedure discussed above. The films were prepared to ensure the density in the middle of the film agreed with the bulk density of the model [8], and they contained typically 2400 ion pairs, with a wall to wall distance of the order of 13 nm. We calculated the potential drop across the interfaces for surface charge in the range 0-3 $\frac{\mu F}{cm^2}$. We found that the voltage surface charge dependence follows a linear dependence, similar to what is observed in the molecular fluids or reported in previous simulations of ionic liquids [9].

In Supplementary Figure 2, we show the electrostatic potential of the full system, including the substrate and RTIL contributions.

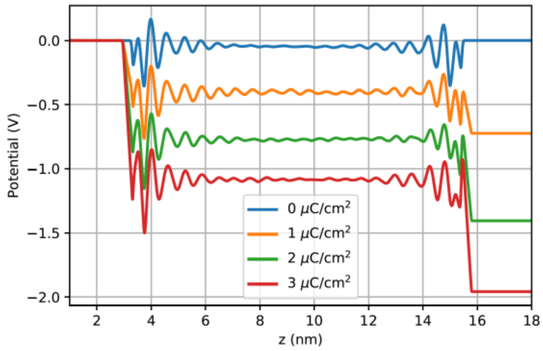

Supplementary Figure 2. The simulated electrostatic potential profile for ionic liquid, including the substrate and RTIL contributions.

Supplementary Note 3: Derivation of Drag Torque Acting on the Discs

Consider a disk of radius R rotating about the z-axis with angular frequency ω. Situated parallel to that disk, at a distance h, is an immobile disk of the same radius R. For $R \gg h$, and after taking advantage of the azimuthal symmetry, we get the Von-Karman differential equations on the velocity components $v_{\mathrm{r}}, v_{\varphi}, v_{\mathrm{z}}$ in cylindrical coordinates:

$$\frac{2v_{\mathrm{r}}}{r} + \frac{dv_{\mathrm{z}}}{dz} = 0 \tag{16}$$

$$\left(\frac{v_{\mathrm{r}}}{r}\right)^2 - \left(\frac{v_{\varphi}}{r}\right)^2 + v_{\mathrm{z}}\frac{d\left(\frac{v_{\mathrm{r}}}{r}\right)}{dz} = -\frac{1}{\rho}\frac{\partial p}{\partial r} + \nu\frac{d^2\left(\frac{v_{\mathrm{r}}}{r}\right)}{dz^2} \tag{17}$$

$$v_{\mathrm{z}}\frac{dv_{\mathrm{z}}}{dz} = -\frac{1}{\rho}\frac{\partial p}{\partial z} + \nu\frac{d^2 v_{\mathrm{z}}}{dz^2} \tag{18}$$

$$\frac{2v_{\mathrm{r}}v_{\varphi}}{r^2} + v_{\mathrm{z}}\frac{d\left(\frac{v_{\varphi}}{r}\right)}{dz} = \nu\frac{d^2\left(\frac{v_{\varphi}}{r}\right)}{dz^2} \tag{19}$$

Where $\frac{v_{\mathrm{r}}}{r}, \frac{v_{\varphi}}{r}$ and v_{z} are functions of z only, and $p = p(r, z)$.

Taking a linear approximation and dropping all non-linear velocity terms:

$$\frac{2v_{\mathrm{r}}}{r} + \frac{dv_{\mathrm{z}}}{dz} = 0 \tag{20}$$

$$-\frac{1}{\rho}\frac{\partial p}{\partial r}+\nu\frac{d^2\left(\frac{v_{\mathrm{r}}}{r}\right)}{dz^2}=0 \tag{21}$$

$$-\frac{1}{\rho}\frac{\partial p}{\partial z}+\nu\frac{d^2 v_{\mathrm{z}}}{dz^2}=0 \tag{22}$$

$$\nu\frac{d^2\left(\frac{v_{\varphi}}{r}\right)}{dz^2}=0 \tag{23}$$

The first three differential equations, (20)- (22), present a coupling between $v_{\mathrm{r}}, v_{\mathrm{z}}$ and p. The fourth equation is uncoupled, and its solution is given by:

$$\frac{v_{\varphi}}{r}=A+Bz \tag{24}$$

Where A and B are constants to be determined by the boundary conditions of the problem. Applying non-slip conditions for the rotating disk at $z=0$ and for the immobile disk is at $z=h$, we get:

$$v_{\varphi}(r,z)=\omega r\left(1-\frac{z}{h}\right) \tag{25}$$

If we introduce a slipping length δ, Supplementary Equation (25) becomes,

$$v_{\varphi}(r,z)=\omega r\left(1-\frac{z}{h+\delta}\right) \tag{26}$$

The shear stress is then given by:

$$\frac{\partial v_{\varphi}}{\partial z}=-\omega r\frac{1}{h+\delta} \tag{27}$$

Consequently, the drag torque is given by,

$$\tau=\int_0^R\int_0^{2\pi}\nu\omega\frac{1}{h+\delta}r^3 dr d\varphi=\frac{1}{2}\pi\nu\omega R^4\frac{1}{h+\delta} \tag{28}$$

For N devices,

$$\tau=\frac{1}{2}\pi\nu\omega R^4\frac{N}{h+\delta} \tag{29}$$

Supplementary Note 4: Derivation of the Expressions for $Q(t)$ and $j(t)$

Consider the electric circuit of our proposed device, as sketched in Supplementary Figure 3.

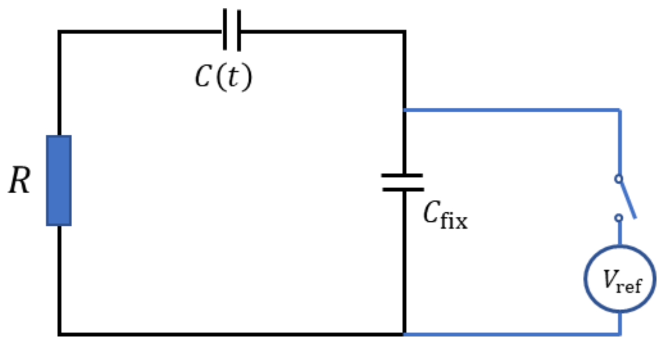

Supplementary Figure 3. A sketch of the electric circuit of our proposed device.

Suppose we momentarily charge C_{fix} using a battery of voltage V_{ref} and then immediately disconnect the voltage source. As a result, one branch of the circuit will possess an excess charge q and the other branch will possess an excess charge $-q$. The total charge in each branch is conserved:

$$Q_{\mathrm{fix}}(t) + Q(t) = q = C_{\mathrm{fix}} V_{\mathrm{ref}} \tag{30}$$

Where $Q_{\mathrm{fix}}(t)$ and $Q(t)$ are the charges in the fixed capacitor and the overall charge on the time-varying capacitors respectively. Kirchhoff's voltage law in the circuit reads:

$$V_{\mathrm{fix}}(t) - V_{\mathrm{R}} - V(t) = 0 \tag{31}$$

Using the relation between the charge on the capacitor and the voltage on its terminals we have,

$$\frac{Q_{\mathrm{fix}}(t)}{C_{\mathrm{fix}}} - Rj(t) - \frac{Q(t)}{C(t)} = 0 \tag{32}$$

Where $j(t)$ is the current in the circuit. Using Supplementary Equation (30) to eliminate Q_{fix}:

$$\frac{C_{\mathrm{fix}} V_{\mathrm{ref}} - Q(t)}{C_{\mathrm{fix}}} - Rj(t) - \frac{Q(t)}{C(t)} = 0 \tag{33}$$

Dividing by $(-R)$ and identifying $j(t) = \frac{dQ}{dt}$, we get,

$$\frac{dQ}{dt} + \frac{Q(t)}{R}\left(\frac{1}{C_{\text{fix}}} + \frac{1}{C(t)}\right) = \frac{V_{\text{ref}}}{R} \tag{34}$$

The term in the brackets of Supplementary Equation (34) is just the equivalent inverse capacitance of the two capacitors connected in series. Since $C_{\text{fix}} \gg C(t)$, we can approximate the equivalent capacitance as that of the time changing capacitor alone. For such approximation we get,

$$\frac{dQ}{dt} + \frac{Q(t)}{RC(t)} = \frac{V_{\text{ref}}}{R} \tag{35}$$

Which is independent of the reservoir capacitance. Let us first introduce the following rescaling parameters:

$$C(t) = NC_{\text{max}}f(t), \qquad \tau \equiv RNC_{\text{max}}, \qquad j_0 \equiv \frac{V_{\text{ref}}}{R} \tag{36}$$

Plugging these into Supplementary Equation (35) we get,

$$\frac{dQ}{dt} + \frac{1}{f(t)}\frac{Q(t)}{\tau} = j_0 \tag{37}$$

This is a first order linear ODE with time-dependent coefficients. The general solution for it is given by,

$$Q(t) = \frac{1}{I(t)}\left[\int_0^t j_0 I(t')dt' + Q(0)\right] \tag{38}$$

where:

$$I(t) \equiv \exp\left[\frac{1}{\tau}\int_0^t \frac{dt'}{f(t')}\right] \tag{39}$$

Initially, the current in the circuit is zero, so that the excess charge distributes between the two capacitors correspond to zero voltage between them. Therefore, initially,

$$\tilde{V}(0) - V(0) = \frac{Q_{\text{fix}}(0)}{C_{\text{fix}}} - \frac{Q(0)}{NC(0)} = \frac{V_{\text{ref}}C_{\text{fix}} - Q(0)}{\tilde{C}} - \frac{Q(0)}{NC(0)} = 0 \tag{40}$$

And so,

$$Q(0) \simeq V_{\text{ref}}NC(0) \tag{41}$$

Plugging Supplementary Equation (41) into Supplementary Equation (38) we get,

$$Q(t) = \frac{1}{I(t)}\left(\int_0^t j_0 I(t')dt' + V_{\mathrm{ref}}C(0)\right) = \frac{j_0}{I(t)}\left(\int_0^t I(t')dt' + RC(0)\right) \tag{42}$$

At this point, to further continue the analysis, we need to model $f(t)$. The capacitance $C(t)$ changes continuously, on each period, from its minimal value, $C_{\min}$, to its maximal value, $C_{\max}$, and back to $C_{\min}$. Therefore, let us model $f(t)$ as:

$$f(t) = \sin^2\left(\frac{\omega t}{2}\right) + \frac{C_{\min}}{C_{\max}}\cos^2\left(\frac{\omega t}{2}\right) \equiv \sin^2\left(\frac{\omega t}{2}\right) + \gamma\cos^2\left(\frac{\omega t}{2}\right) \tag{43}$$

where $\gamma = \frac{C_{\min}}{C_{\max}}$. Substituting Supplementary Equation (43) into Supplementary Equation (39), we get –

$$I(t) \equiv \exp\left[\frac{1}{\tau}\int_0^t \frac{dt'}{\sin^2\left(\frac{\omega t}{2}\right) + \gamma\cos^2\left(\frac{\omega t}{2}\right)}\right] \tag{44}$$

Rearranging Supplementary Equation (44) by using trigonometric identities we arrive at:

$$I(t) = \exp\left[\frac{1}{\tau}\int_0^t \frac{dt'}{\frac{1+\gamma}{2} - \frac{1-\gamma}{2}\cos(\omega t')}\right] \tag{45}$$

Luckily, this class of integrals has a closed-form solution,

$$\int_0^x \frac{dx'}{p + q\cos x'} = \frac{2\left(\arctan\left[\sqrt{\frac{p-q}{p+q}}\tan\left(\frac{x}{2}\right)\right] + \pi * \mathrm{Floor}\left[\frac{x+\pi}{2\pi}\right]\right)}{\sqrt{p^2 - q^2}} \quad ; p > q \tag{46}$$

where the function $\mathrm{Floor}[\eta] \equiv \eta - \left(\frac{1}{2} + \frac{\arctan\left(\tan\left(\pi\left(\eta + \frac{1}{2}\right)\right)\right)}{\pi}\right)$, returns the greatest integer less than or equal to η. And so,

$$I(t) = \exp\left[\frac{2}{\omega\sqrt{\gamma}\tau}\left(\arctan\left[\sqrt{\frac{1}{\gamma}}\tan\left(\frac{\omega t}{2}\right)\right] + \pi * \mathrm{Floor}\left[\frac{\omega t + \pi}{2\pi}\right]\right)\right] \tag{47}$$

Plugging Supplementary Equation (47) into Supplementary Equation (42), and using the identity $\arctan x - \arctan y = \arctan\left(\frac{x-y}{1+xy}\right)$, we get the final expression for the charge:

$$Q(t) = \exp\left[-\frac{2}{\omega\sqrt{\gamma}\tau}\arctan\left(\frac{\left(\sqrt{\frac{1}{\gamma}}-1\right)\tan\left(\frac{\omega t}{2}\right)}{1+\sqrt{\frac{1}{\gamma}}\tan^2\left(\frac{\omega t}{2}\right)}\right)\right]$$

$$\times\left(j_0\int_0^t \exp\left(-\frac{t-t'}{\sqrt{\gamma}\tau}\right)\exp\left[\frac{2}{\omega\sqrt{\gamma}\tau}\arctan\left(\frac{\left(\sqrt{\frac{1}{\gamma}}-1\right)\tan\left(\frac{\omega t'}{2}\right)}{1+\sqrt{\frac{1}{\gamma}}\tan^2\left(\frac{\omega t'}{2}\right)}\right)\right]dt' \right. \quad (48)$$

$$\left. + V_{\mathrm{ref}}\gamma\bar{C}\exp\left(-\frac{t}{\sqrt{\gamma}\tau}\right)\right)$$

where $\tau = RNC_{\max}$.

Supplementary Note 5: Stationary Expressions for $Q(t)$, $j(t)$ and Average Power in the High Frequency Regime

The steady state regime is when initial conditions memory disappears. This happens at times $t \gg \sqrt{\gamma}\tau$. The steady state solution for the overall charge on the time-varying capacitors is:

$$Q_{\mathrm{ss}}(t) = j_0\int_0^t \exp\left(-\frac{t-t'}{\sqrt{\gamma}\tau}\right)\exp\left[\frac{2}{\omega\sqrt{\gamma}\tau}\left(\arctan\left(\frac{\left(\sqrt{\frac{1}{\gamma}}-1\right)\tan\left(\frac{\omega t'}{2}\right)}{1+\sqrt{\frac{1}{\gamma}}\tan^2\left(\frac{\omega t'}{2}\right)}\right) - \arctan\left(\frac{\left(\sqrt{\frac{1}{\gamma}}-1\right)\tan\left(\frac{\omega t}{2}\right)}{1+\sqrt{\frac{1}{\gamma}}\tan^2\left(\frac{\omega t}{2}\right)}\right)\right)\right]dt' \quad (49)$$

Using $\arctan x - \arctan y = \arctan\left(\frac{x-y}{1+xy}\right)$

$$Q_{\mathrm{ss}}(t)$$

$$= j_0 \int_0^t \exp\left(-\frac{t-t'}{\sqrt{\gamma}\tau}\right) \exp\left[\frac{2}{\omega\sqrt{\gamma}\tau}\arctan\left(\frac{\frac{\left(\sqrt{\frac{1}{\gamma}}-1\right)\tan\left(\frac{\omega t'}{2}\right)}{1+\sqrt{\frac{1}{\gamma}}\tan^2\left(\frac{\omega t'}{2}\right)} - \frac{\left(\sqrt{\frac{1}{\gamma}}-1\right)\tan\left(\frac{\omega t}{2}\right)}{1+\sqrt{\frac{1}{\gamma}}\tan^2\left(\frac{\omega t}{2}\right)}}{1+\frac{\left(\sqrt{\frac{1}{\gamma}}-1\right)\tan\left(\frac{\omega t'}{2}\right)\left(\sqrt{\frac{1}{\gamma}}-1\right)\tan\left(\frac{\omega t}{2}\right)}{1+\sqrt{\frac{1}{\gamma}}\tan^2\left(\frac{\omega t'}{2}\right)\ 1+\sqrt{\frac{1}{\gamma}}\tan^2\left(\frac{\omega t}{2}\right)}}\right)\right] dt' \tag{50}$$

For high frequencies, $\omega \gg \frac{1}{\sqrt{\gamma}\tau}$, we can expand the second exponential term to a leading term:

$$Q_{\mathrm{ss}}(t) \simeq j_0 \int_0^t \exp\left(-\frac{t-t'}{\sqrt{\gamma}\tau}\right) \times \left(1+\frac{2}{\omega\sqrt{\gamma}\tau}\arctan\left(\frac{\frac{\left(\sqrt{\frac{1}{\gamma}}-1\right)\tan\left(\frac{\omega t'}{2}\right)}{1+\sqrt{\frac{1}{\gamma}}\tan^2\left(\frac{\omega t'}{2}\right)} - \frac{\left(\sqrt{\frac{1}{\gamma}}-1\right)\tan\left(\frac{\omega t}{2}\right)}{1+\sqrt{\frac{1}{\gamma}}\tan^2\left(\frac{\omega t}{2}\right)}}{1+\frac{\left(\sqrt{\frac{1}{\gamma}}-1\right)\tan\left(\frac{\omega t'}{2}\right)\left(\sqrt{\frac{1}{\gamma}}-1\right)\tan\left(\frac{\omega t}{2}\right)}{1+\sqrt{\frac{1}{\gamma}}\tan^2\left(\frac{\omega t'}{2}\right)\ 1+\sqrt{\frac{1}{\gamma}}\tan^2\left(\frac{\omega t}{2}\right)}}\right)\right) dt' \tag{51}$$

or, simply,

$$Q_{\mathrm{ss}}(t) = Q_1(t) + Q_2(t) \tag{52}$$

where,

$$\begin{aligned} Q_1(t) &= j_0 \int_0^t \exp\left(-\frac{t-t'}{\sqrt{\gamma}\tau}\right) dt' = j_0\sqrt{\gamma}\tau\left(1-e^{-\frac{t}{\sqrt{\gamma}\tau}}\right) \\ &= \sqrt{\gamma}V_{\mathrm{ref}}C_{\max}\left(1-e^{-\frac{t}{\sqrt{\gamma}\tau}}\right) \approx \sqrt{\gamma}V_{\mathrm{ref}}C_{\max} \end{aligned} \tag{53}$$

for steady state large times, and,

$$Q_2(t) = \frac{2j_0}{\omega\sqrt{\gamma}\tau}\int_0^t \exp\left(-\frac{t-t'}{\sqrt{\gamma}\tau}\right)\arctan\left(\frac{\frac{\left(\sqrt{\frac{1}{\gamma}}-1\right)\tan\left(\frac{\omega t'}{2}\right)}{1+\sqrt{\frac{1}{\gamma}}\tan^2\left(\frac{\omega t'}{2}\right)} - \frac{\left(\sqrt{\frac{1}{\gamma}}-1\right)\tan\left(\frac{\omega t}{2}\right)}{1+\sqrt{\frac{1}{\gamma}}\tan^2\left(\frac{\omega t}{2}\right)}}{1+\frac{\left(\sqrt{\frac{1}{\gamma}}-1\right)\tan\left(\frac{\omega t'}{2}\right)\left(\sqrt{\frac{1}{\gamma}}-1\right)\tan\left(\frac{\omega t}{2}\right)}{1+\sqrt{\frac{1}{\gamma}}\tan^2\left(\frac{\omega t'}{2}\right)\ 1+\sqrt{\frac{1}{\gamma}}\tan^2\left(\frac{\omega t}{2}\right)}}\right) dt' \tag{54}$$

This analysis shows that the total charge $Q_{\mathrm{ss}}(t)$, on the time-varying capacitor in steady state, is given by the sum of $Q_1 \approx \sqrt{\gamma}V_{\mathrm{ref}}C_{\max}$, which is constant in time and independent of the rotation frequency ω, and $Q_2(t)$ which is an oscillatory function whose magnitude rapidly decreases with increasing ω. This is evident from Supplementary Figure 4, which shows the numerical solution for the charge as function of time at various high frequencies.

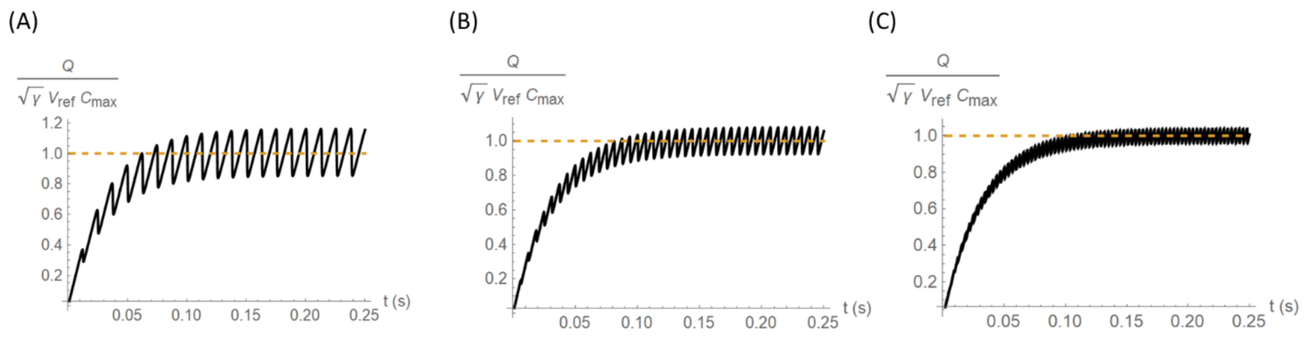

Supplementary Figure 4. The charge on the time varying capacitor as function of time for system parameters: $V_{\mathrm{ref}} = 50\ \mathrm{V},\ \tau = 1\ \mathrm{s}, \gamma = 10^{-3}$ and for angular frequency: (A) $\omega = 500\ \mathrm{s}^{-1}$ (B) $\omega = 1000\ \mathrm{s}^{-1}$ (C) $\omega = 2000\ \mathrm{s}^{-1}$.

The steady state current is given by:

$$\frac{dQ_{ss}}{dt} \approx \frac{dQ_2}{dt} \tag{55}$$

which is given by,

$$\frac{dQ_2}{dt} = \frac{2j_0}{\omega\sqrt{\gamma}\tau}\frac{d}{dt}\int_0^t \exp\left(-\frac{t-t'}{\sqrt{\gamma}\tau}\right)\arctan\left(\frac{\dfrac{\left(\sqrt{\frac{1}{\gamma}}-1\right)\tan\left(\frac{\omega t'}{2}\right)}{1+\sqrt{\frac{1}{\gamma}}\tan^2\left(\frac{\omega t'}{2}\right)} - \dfrac{\left(\sqrt{\frac{1}{\gamma}}-1\right)\tan\left(\frac{\omega t}{2}\right)}{1+\sqrt{\frac{1}{\gamma}}\tan^2\left(\frac{\omega t}{2}\right)}}{1+\dfrac{\left(\sqrt{\frac{1}{\gamma}}-1\right)\tan\left(\frac{\omega t'}{2}\right)}{1+\sqrt{\frac{1}{\gamma}}\tan^2\left(\frac{\omega t'}{2}\right)}\dfrac{\left(\sqrt{\frac{1}{\gamma}}-1\right)\tan\left(\frac{\omega t}{2}\right)}{1+\sqrt{\frac{1}{\gamma}}\tan^2\left(\frac{\omega t}{2}\right)}}\right)dt' \tag{56}$$

requires us to differentiate under the integral sign (Leibniz rule). This will result in two additive terms, one with differentiation of the upper limit, and the other with differentiation of the integrand. The former's amplitude still goes like $\frac{1}{\omega}$ and therefore vanishes at high ω. The latter term has a non-vanishing amplitude and is given by:

$$\frac{2j_0}{\omega\sqrt{\gamma}\tau}\int_0^t \frac{d}{dt}\left(\exp\left(-\frac{t-t'}{\sqrt{\gamma}\tau}\right)\arctan\left(\frac{\dfrac{\left(\sqrt{\frac{1}{\gamma}}-1\right)\tan\left(\frac{\omega t'}{2}\right)}{1+\sqrt{\frac{1}{\gamma}}\tan^2\left(\frac{\omega t'}{2}\right)} - \dfrac{\left(\sqrt{\frac{1}{\gamma}}-1\right)\tan\left(\frac{\omega t}{2}\right)}{1+\sqrt{\frac{1}{\gamma}}\tan^2\left(\frac{\omega t}{2}\right)}}{1+\dfrac{\left(\sqrt{\frac{1}{\gamma}}-1\right)\tan\left(\frac{\omega t'}{2}\right)}{1+\sqrt{\frac{1}{\gamma}}\tan^2\left(\frac{\omega t'}{2}\right)}\dfrac{\left(\sqrt{\frac{1}{\gamma}}-1\right)\tan\left(\frac{\omega t}{2}\right)}{1+\sqrt{\frac{1}{\gamma}}\tan^2\left(\frac{\omega t}{2}\right)}}\right)\right)dt' \tag{57}$$

This derivative will result with two additive terms. The first's amplitude will still decay with ω, but the second term's amplitude will be independent of ω, as we shall show here.

$$\frac{dQ_2}{dt} \approx \frac{2j_0}{\sqrt{\gamma}\tau}\int_0^t \exp\left(-\frac{t-t'}{\sqrt{\gamma}\tau}\right)\frac{d}{d(\omega t)}\arctan\left(\frac{\frac{\left(\sqrt{\frac{1}{\gamma}}-1\right)\tan\left(\frac{\omega t'}{2}\right)}{1+\sqrt{\frac{1}{\gamma}}\tan^2\left(\frac{\omega t'}{2}\right)}-\frac{\left(\sqrt{\frac{1}{\gamma}}-1\right)\tan\left(\frac{\omega t}{2}\right)}{1+\sqrt{\frac{1}{\gamma}}\tan^2\left(\frac{\omega t}{2}\right)}}{1+\frac{\left(\sqrt{\frac{1}{\gamma}}-1\right)\tan\left(\frac{\omega t'}{2}\right)\left(\sqrt{\frac{1}{\gamma}}-1\right)\tan\left(\frac{\omega t}{2}\right)}{\left(1+\sqrt{\frac{1}{\gamma}}\tan^2\left(\frac{\omega t'}{2}\right)\right)\left(1+\sqrt{\frac{1}{\gamma}}\tan^2\left(\frac{\omega t}{2}\right)\right)}}\right)dt' \tag{58}$$

Let us define:

$$x \equiv \omega t, y \equiv \omega t', f(x,y) \equiv \arctan\left(\frac{\frac{\left(\sqrt{\frac{1}{\gamma}}-1\right)\tan\left(\frac{y}{2}\right)}{1+\sqrt{\frac{1}{\gamma}}\tan^2\left(\frac{y}{2}\right)}-\frac{\left(\sqrt{\frac{1}{\gamma}}-1\right)\tan\left(\frac{x}{2}\right)}{1+\sqrt{\frac{1}{\gamma}}\tan^2\left(\frac{x}{2}\right)}}{1+\frac{\left(\sqrt{\frac{1}{\gamma}}-1\right)\tan\left(\frac{y}{2}\right)\left(\sqrt{\frac{1}{\gamma}}-1\right)\tan\left(\frac{x}{2}\right)}{\left(1+\sqrt{\frac{1}{\gamma}}\tan^2\left(\frac{y}{2}\right)\right)\left(1+\sqrt{\frac{1}{\gamma}}\tan^2\left(\frac{x}{2}\right)\right)}}\right), \tag{59}$$

$$g(x,y) \equiv \frac{df(x,y)}{dx}.$$

The steady state current is now given by:

$$j_{\mathrm{ss}}(t) = \frac{2j_0}{\sqrt{\gamma}\tau\omega}\int_0^x g(x,y)\exp\left(-\frac{x-y}{\sqrt{\gamma}\tau\omega}\right)dy \tag{60}$$

Using integration by parts:

$$j_{\mathrm{ss}}(t) = 2j_0\left(\left[g(x,x)-g(x,0)\exp\left(-\frac{x}{\sqrt{\gamma}\tau\omega}\right)\right]-\int_0^x \exp\left(-\frac{x-y}{\sqrt{\gamma}\tau\omega}\right)\frac{dg}{dy}dy\right) \tag{61}$$

However, $g(x,0)\exp\left(-\frac{x}{\sqrt{\gamma}\tau\omega}\right) \approx 0$ in the steady state by the definition of the steady state times. Therefore, we are left with:

$$j_{\mathrm{ss}} = 2j_0\left(g(x,x)-\int_0^x \exp\left(-\frac{x-y}{\sqrt{\gamma}\tau\omega}\right)\frac{dg}{dy}dy\right) \tag{62}$$

Since $\frac{dg}{dy}$ is a periodic function which is symmetric about $y = 0$, and because the function $\exp\left(-\frac{x-y}{\sqrt{\gamma}\tau\omega}\right)$ is a very slowly decaying envelope, then the integral in Supplementary Equation (62) sums over approximately equal negative and positive contributions and is therefore negligible. Thereby, we are left with:

$$j_{\mathrm{ss}} = 2j_0 g(x,x) \tag{63}$$

By comparison of $2j_0 g(x,x)$ and the steady state current given solved numerically, we get approximately the same profile. Calculating $g(x,x)$ explicitly we get,

$$j_{\mathrm{ss}}(t) = j_0\left(1 - \frac{2\sqrt{\gamma}}{1+\gamma-(1-\gamma)\cos(\omega t)}\right) \tag{64}$$

By plugging Supplementary Equation (64) into the average power formula given by Equation (14) in the main text, we can acquire the average power P_0 at the regime of high rotational frequencies $\omega \gg \frac{1}{\sqrt{\gamma}\tau}$:

$$P_0 = j_0^2 R\frac{(\sqrt{\gamma}-1)^2}{2\sqrt{\gamma}} = \frac{V_{\mathrm{ref}}^2}{R}\frac{(1-\sqrt{\gamma})^2}{2\sqrt{\gamma}} \tag{65}$$

Supplementary Note 6: Full Analysis of the Performance Frequencies

The stationary performance frequency of the rotor is determined by the power balance:

$$\tau_{\mathrm{p}}\omega - \tau_{\mathrm{d}}\omega - P_{\mathrm{R}} = 0 \tag{66}$$

where, in the main text, P_{R} is given by Equation (14), τ_{d} is the drag torque given by Equation (17) and τ_{p} is the generative press torque given by Equation (15). Plugging in the expressions for τ_{d}, τ_{p} and P_{R}, and dividing Supplementary Equation (66) by ωr, where r is the radius of the wheel, we get:

$$F_{\mathrm{p}} = \frac{1}{2}\pi\nu\omega r^3\frac{N}{l+\delta} + \frac{\int_0^{2\pi/\omega} Rj^2(t)dt}{2\pi r} \tag{67}$$

Where $F_{\mathrm{p}} \approx 25$ N is the force of foot press averaged over the period of a foot press (~1 s). To determine ω from this transcendental equation, we calculated the right-hand side of Supplementary Equation (67) for different ω and plotted it in Supplementary Figure 5. The

intersection point with the constant F_p will give the suitable performance frequency of the rotor. Moreover, to check the validity of the approximation made in the main text, where we calculate ω by equating the drag torque to the generative torque, we plotted a second curve containing only the viscous drag term in Supplementary Equation (67).

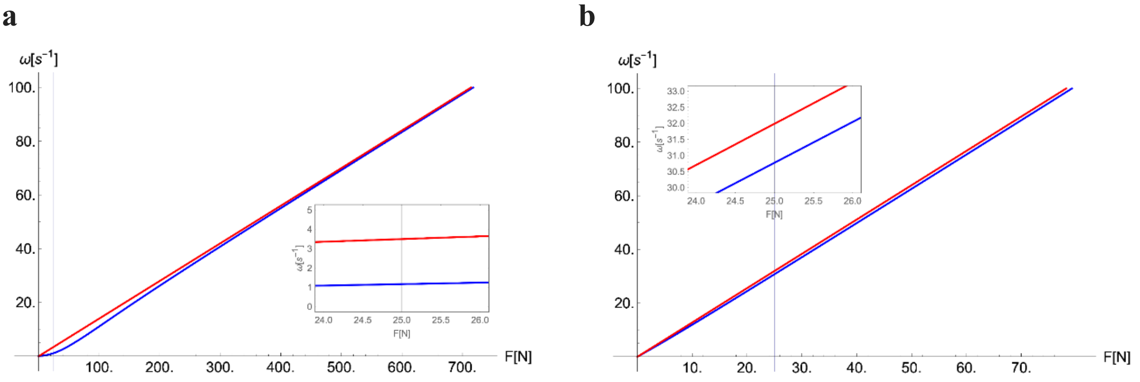

Supplementary Figure 5. The angular frequency as a function of the drag force (red curve) and as a function of the sum of drag force together with the resistive load effective force. The vertical line represents the average force of press F_p. Subfigures **(a)** and **(b)** correspond to two different rotor setups:

(a) PC lubricant with $\nu = 2.5$ mPa s, $N = 10^2$, $r = 10^{-2}$ m, $\delta = 50$ nm and $l = 5$ nm.

(b) IL mixture lubricant with $\nu = 5$ mPa s, $N = 10^3$, $r = 10^{-2}$ m and $l = 10$ µm.

As seen in Supplementary Figure 5, the difference between the two curves becomes smaller as ω increases. The approximation made in the main text is within reason in the case of PC, and completely valid in the case of IL mixtures.

Supplementary References

1. Bussi, G., Donadio, D. & Parrinello, M. Canonical sampling through velocity rescaling. *J. Chem. Phys.* **126**, 014101 (2007).

2. Berendsen, H. J. C., Postma, J. P. M., van Gunsteren, W. F., DiNola, A. & Haak, J. R. Molecular dynamics with coupling to an external bath. *J. Chem. Phys.* **81**, 3684–3690 (1984).

3. Yeh, I.-C. & Berkowitz, M. L. Ewald summation for systems with slab geometry. *J. Chem. Phys.* **111**, 3155–3162 (1999).

4. Besler, B. H., Merz, K. M. & Kollman, P. A. Atomic charges derived from semiempirical methods. *J. Comput. Chem.* **11**, 431–439 (1990).

5. Singh, U. C. & Kollman, P. A. An approach to computing electrostatic charges for molecules. *J. Comput. Chem.* **5**, 129–145 (1984).

6. Daniels, I. N., Wang, Z. & Laird, B. B. Dielectric Properties of Organic Solvents in an Electric Field. *J. Phys. Chem. C* **121**, 1025–1031 (2017).

7. Yang, L., Fishbine, B. H., Migliori, A. & Pratt, L. R. Dielectric saturation of liquid propylene carbonate in electrical energy storage applications. *J. Chem. Phys.* **132**, 044701 (2010).

8. Fajardo, O. Y., Di Lecce, S. & Bresme, F. Molecular dynamics simulation of imidazolium C n MIM-BF 4 ionic liquids using a coarse grained force-field. *Phys. Chem. Chem. Phys.* **22**, 1682–1692 (2020).

9. Merlet, C., Salanne, M. & Rotenberg, B. New Coarse-Grained Models of Imidazolium Ionic Liquids for Bulk and Interfacial Molecular Simulations. *J. Phys. Chem. C* **116**, 7687–7693 (2012).
